# Photochemistry of Sodium Pyruvate Clusters

**DOI:** 10.1021/acs.jpca.6c02410

**Published:** 2026-06-16

**Authors:** Sarah J. Madlener, Marc Reimann, Jessica C. Hartmann, Christian van der Linde, Milan Ončák, Martin K. Beyer

**Affiliations:** Universität Innsbruck, Institut für Ionenphysik und Angewandte Physik, Technikerstraße 25, Innsbruck 6020, Austria

## Abstract

Pyruvate is frequently found in aged sea salt aerosols. To test the influence of the salt environment on the photochemistry of pyruvate, small model clusters were generated from sodium pyruvate salt using electrospray ionization. These clusters were studied using Fourier-Transform Ion Cyclotron Resonance mass spectrometry (FT-ICR MS). UV/vis spectra were recorded using a tunable optical parametric oscillator (OPO) system. Additionally, photokinetics as well as sustained off-resonance irradiation (SORI) collision-induced dissociation (CID) experiments help to further understand the mechanisms responsible for the fragmentation of the studied systems. Quantum chemical calculations allow assignment of the transitions responsible for the absorptions. The clusters mainly fragment by losing sodium pyruvate units, Na*_m_* Pyr*_m_*, resulting in Na_*n*–*m*_Pyr_*n*–*m*–1_^+^ fragments. Photodissociation cross sections for nonstoichiometric fragmentation are around 2 orders of magnitude lower. We found that the formation of these fragments starts with the C–C bond photolysis of a pyruvate anion and can lead to several secondary fragments. For the two larger cluster sizes, neutral CH_3_COCOO is lost, presumably in the form of a CH_3_CO radical, followed by CO_2_ elimination. The electron is transferred to a second pyruvate molecule in the cluster, resulting in a radical dianion, CH_3_COCOO^2–^. For the smallest cluster size, a variety of secondary fragments was observed, including CO_2_^–^ stabilized by two Na^+^ ions in the cluster.

## Graphical abstract

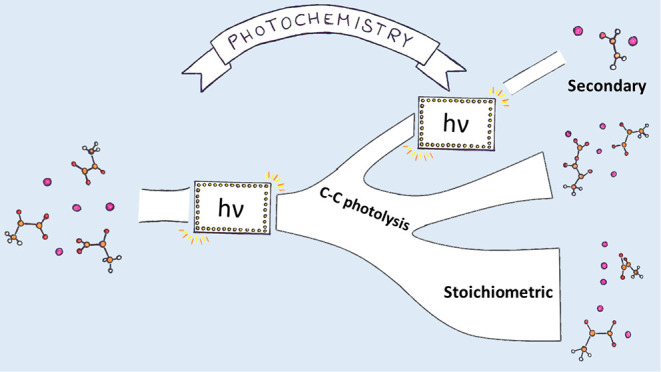

## Introduction

Pyruvic acid, as well as its conjugate base pyruvate, is ubiquitous in the atmosphere. Both are produced naturally and from anthropogenic sources. In contrast to many other volatile organic compounds (VOCs), which are predominantly oxidized by OH radicals,^1^ pyruvic acid in the atmosphere mainly undergoes photolysis^2,3^ via an (*n*,π*) electronic transition with a maximum around 350 nm,^4^ which may in turn trigger subsequent chemical reactions, such as oligomerization.^5^ The absorption maximum of this transition thus lies in the actinic region, which describes the wavelength range of solar radiation from 290–420 nm, which reaches the troposphere and has sufficient energy for photochemical transformations.^6,7^

Pyruvic acid photolysis in the gas phase has been investigated over a wide range of experimental conditions and described through various quantum chemical methods. Early studies reported the photolysis of pyruvic acid to produce CO_2_ and acetaldehyde^8–10^ via decarboxylation through a cyclic transition state. Mellouki and Mu reported an absorption in the actinic region with a maximum around 350 nm, in agreement with previous studies.^11^ A series of experiments addressed the photodissociation dynamics, focusing on the energy distribution of pyruvic acid photofragments, especially CO_2_.^12–17^ The recent velocity map imaging (VMI) work by Reisler, Osborn and co-workers provides a very detailed picture of the photofragments at 193 nm, including CO_2_, CO, H, OH, HCO, CH_2_CO, CH_3_CO, and CH_3_.^15^ Very recently, Sauer and Davis identified three competing photodissociation pathways of pyruvic acid in a molecular beam for selected wavelengths between 320–370 nm,^18^ C–C dissociation on the triplet surface yielding CH_3_CO together with HOCO, and decarboxylation on both triplet and singlet surfaces. The adiabatic singlet–triplet gap in the first electronically excited state was determined by Verlet and co-workers as 0.29 ± 0.04 eV by photoelectron spectroscopy.^19^ A VUV study by Poisson, Hochlaf, and co-workers provided further insight into the dynamics of decarboxylation for CH_3_COCOOH and CH_3_COCOOH^+^.^20^ Quantum chemical calculations were performed for pyruvic acid and its photochemistry to investigate the bond dynamics, structure, and decomposition, as well as the photochemical mechanism of decarboxylation.^21–26^

Besides photochemistry of isolated molecules under vacuum conditions, gas-phase studies of pyruvic acid cover topics such as reactions with HO_2_,^27^ rotational spectroscopy of the pyruvic acid–water complex,^28^ and the photochemical decomposition of pyruvic acid in air.^29^ In aqueous solution, the properties and the mechanisms of the photolysis, as well as secondary reactions of pyruvic acid, were studied by UV/vis and NMR spectroscopy^30–36^ as well as high-performance liquid chromatography (HPLC) combined with electrospray ionization mass spectrometry (ESI-MS).^37,38^ Vaida’s group has systematically studied the photochemistry of pyruvic acid under conditions relevant to atmospheric chemistry, like the gas phase,^39,40^ aqueous phase,^41–46^ and the air–water interface.^47,48^

In aged sea salt aerosols, pyruvate anions rather than pyruvic acid are frequently found.^49–51^ The concomitant depletion of chloride in the clusters indicates that acid displacement has occurred,^50–54^ which means that the aerosols actually contain sodium pyruvate rather than dissolved pyruvic acid. In contrast to the numerous studies on pyruvic acid, the photochemistry of pyruvate, its conjugate base, has received less attention. In an early study, photolysis of aqueous sodium pyruvate was found to be slow, and carbon dioxide evolution was not observed.^55^ However, other potential products were not analyzed.^55^ Two studies investigated the photoelectron spectra of pyruvate. Verlet and co-workers found that photoexcitation of gas-phase pyruvate using UVA light leads to C–C bond cleavage, producing CH_3_CO^–^, which further decays into CO and CH_3_^–^.^56^ They also reported that the CH_3_^–^ molecular anion subsequently detaches a low-energy electron. Wang and co-workers^57^ showed that after the addition of two water molecules, all photodissociation channels for pyruvate were blocked. In the absence of photons, energetic collisions may also cause C–C bond cleavage, as observed by Miller and Uggerud in their collision-induced dissociation (CID) mass spectra of CH_3_COCOO^–^ in the gas phase.^58^ Recent ultrafast spectroscopy of aqueous pyruvate by Jensen and co-workers showed that decarboxylation is efficient at 200 nm, but no products were observed after irradiation at 340 nm.^59^ In an experiment motivated by the organic chemistry potentially occurring on the dwarf planet Ceres, Weber et al. observed the release of CO_2_ from macroscopic sodium pyruvate samples irradiated with a xenon arc lamp under vacuum.^60^ All these studies show that pyruvate photochemistry involves C–C bond cleavage, but the details depend sensitively on the environment.

We have previously studied the photochemistry of glyoxylate in dry and hydrated sodium chloride clusters,^6,61^ providing insights into the influence of the salt environment on the photochemistry of glyoxylate. Photolysis of glyoxylate leads to salt clusters containing a stabilized carbon dioxide radical anion, CO_2_^–^, or an HCOO^–^ entity. To learn more about pyruvate photolysis, we here investigated the photochemistry of sodium pyruvate clusters using a Fourier-Transform Ion Cyclotron Resonance mass spectrometer (FT-ICR MS). The UV/vis photodissociation spectra of [Na_*n*_(CH_3_COCOO)_(*n*−1)_]^+^, *n* = 2,4,5 (now labeled as Na_*n*_Pyr_(*n*–1)_^+^) were recorded, and quantitative, product-specific photodissociation cross sections were obtained. Laser photodissociation kinetics and sustained off-resonance irradiation collision-induced dissociation (SORI-CID) measurements provided additional insights. Quantum chemical calculations of the structure and thermochemistry of the photofragments were performed, and the absorption spectra were simulated for comparison with the experimental results.

## Experimental and Theoretical Methods

The experiments are conducted on a Bruker APEX Qe 9.4 T Fourier-Transform Ion Cyclotron Resonance mass spectrometer. The sodium pyruvate clusters are produced using an electrospray ionization source, and the resulting molecular beam is guided toward the ICR cell via two funnels, a first hexapole, a quadrupole mass filter, a second hexapole collision cell, and an electrostatic lens system. For the sample, a 10 mmol L^–1^ solution of sodium pyruvate (≥99%, Roth) was prepared in a 1:1 mixture of water (HPLC grade, Roth) and methanol (HPLC grade, Roth). To perform photodissociation spectroscopy, light from a tunable optical parametric oscillator (OPO) system (EKSPLA NT342B, pulse length 3–5 ns, 20 Hz) is guided through two CaF_2_ prisms and a CaF_2_ window into the ICR cell. The laser power is monitored after each recorded wavelength. A more detailed description of the experimental setup can be found elsewhere.^62^

Depending on the wavelength region, irradiation times of 2 s (in the range of 220–300 nm) or 20 s (in the range of 284–410 nm) were used. The total photodissociation cross section σ_tot_ was obtained using eq 1, using the intensity *I*_0_ of the precursor and *I*_*i*_ of the fragments, *i* > 0, the pulse energy of the laser *E* at wavelength λ, the beam diameter *A* entering the ICR cell, approximated to be 1.25 cm^2^, and the number of laser pulses *p*. No correction for the influence of blackbody infrared radiative dissociation (BIRD)^63^ was applied, as these clusters did not exhibit dissociation without laser irradiation. For background correction, the residual fragment ion intensity that was present without laser irradiation was subtracted from *I*_*i*_ before applying eq 1. For each wavelength, 20 spectra were recorded and averaged.

1 $$\sigma_{\mathrm{tot}}=\frac{\mathrm{hcA}}{\lambda \mathrm{pE}}\left(\ln\frac{\sum_{i=0}^{n}I_{i}}{I_{0}}\right)$$

The dominant contribution to the uncertainty in the photodissociation cross sections is the overlap of the ion cloud with the laser beam. The location of the ion cloud in the center of a narrow-bore superconducting magnet makes precise measurements impossible. However, the photodissociation kinetics shown in Figures S1 and S2 indicate an overlap of at least 50%. Also, the photon flux must be extrapolated from the location of the laser pulse energy measurement close to the laser to the center of the magnet. The overall error for the absolute values of the cross sections is therefore estimated to be around 30%, based on the repeatability of the measurement. The detection of the fragments, however, is independent of error propagation and is not affected by these limitations.

For comparison with photodissociation, SORI-CID experiments were performed using argon (99.999%) as a collision gas. The pressure was kept constant throughout all the CID experiments at 7.5(5) × 10^–9^ mbar. An excitation pulse length of 1 s was used with a frequency offset of 500 Hz. By changing a parameter called “SORI Power” in the software of the instrument (Compass apexControl 3.0.0), the average ion kinetic energy can be modified in a reproducible way, but not quantitatively. In particular, the average kinetic energy as well as the width of its distribution increases nonlinearly with the SORI Power parameter. Therefore, only qualitative information can be obtained from the fragment intensities as a function of SORI Power.

Geometry optimization and frequency calculations were performed at the density functional level using Gaussian (Gaussian 16, revision A03).^64^ All calculations employed the ωB97XD functional^65^ and an aug-cc-pVDZ basis set.^66^ The structures of the clusters (Na_2_Pyr^+^, Na_3_Pyr_2_^+^, Na_3_Pyr_3_, Na_4_Pyr_2_CO_2_^+^, Na_4_Pyr_3_^+^, Na_5_Pyr_4_^+^) were sampled using an in-house genetic algorithm code^67^ employing the semiempirical extended tight-binding (xTB) scheme with the GFN2 Hamiltonian,^68^ and a population of 40 structures and 20 cycles with 20 recombinations in each. The most stable structures in which the Pyr units remained intact were then reoptimized at the DFT level. The radical ions of Na_4_Pyr_2_^+^ and Na_5_Pyr_3_^+^ were optimized after removing a pyruvate unit from Na_4_Pyr_3_^+^ and Na_5_Pyr_4_^+^, respectively. Electronic dipole transitions were calculated using time-dependent DFT in the ORCA program package, version 6.1.1,^69^ employing the ωB97XD functional and the def2-TZVPPD basis set.^70^ The functional was called via the LibXC^71^ interface, and all D2 contributions were neglected, as they do not contribute to the vertical spectra. Both singlet and triplet excited states were considered. Spin–orbit coupling was accounted for by the default mean-field operator (RI-SOMF). The TDDFT results were converted to absorption cross sections using the Multiwfn code.^72,73^

## Results and Discussion

The fragment-resolved photodissociation spectra of Na_*n*_Pyr_*n*–1_^+^, *n* = 4,5, are shown in Figure 1. The photodissociation cross section reaches 5 × 10^–18^ cm^2^ at 220 nm, and gradually falls below 10^–18^ cm^2^ toward 280 nm; see panels (a) and (d) for *n* = 4 and 5, respectively. In the actinic region, λ > 290 nm, the absorption cross section is significantly reduced, as observed previously for glyoxylate.^6,61^ Therefore, an irradiation time of 20 s was used to obtain a sufficient signal-to-noise level of the photofragments. The absorption cross section reaches a local maximum close to 5 × 10^–19^ cm^2^ and levels off beyond 360 nm. The exact values change somewhat with cluster size, as shown in Figure 1b,e, but the overall behavior is quite similar. In particular, a local maximum at 310 nm and a weak structure indicate that several electronic transitions act together to generate the spectrum. This is not surprising, as the chromophore is pyruvate, and the individual molecular ions are embedded in their specific environment in the cluster, which modifies the contribution of each pyruvate ion to the photodissociation spectrum of the cluster.

**Figure 1:**
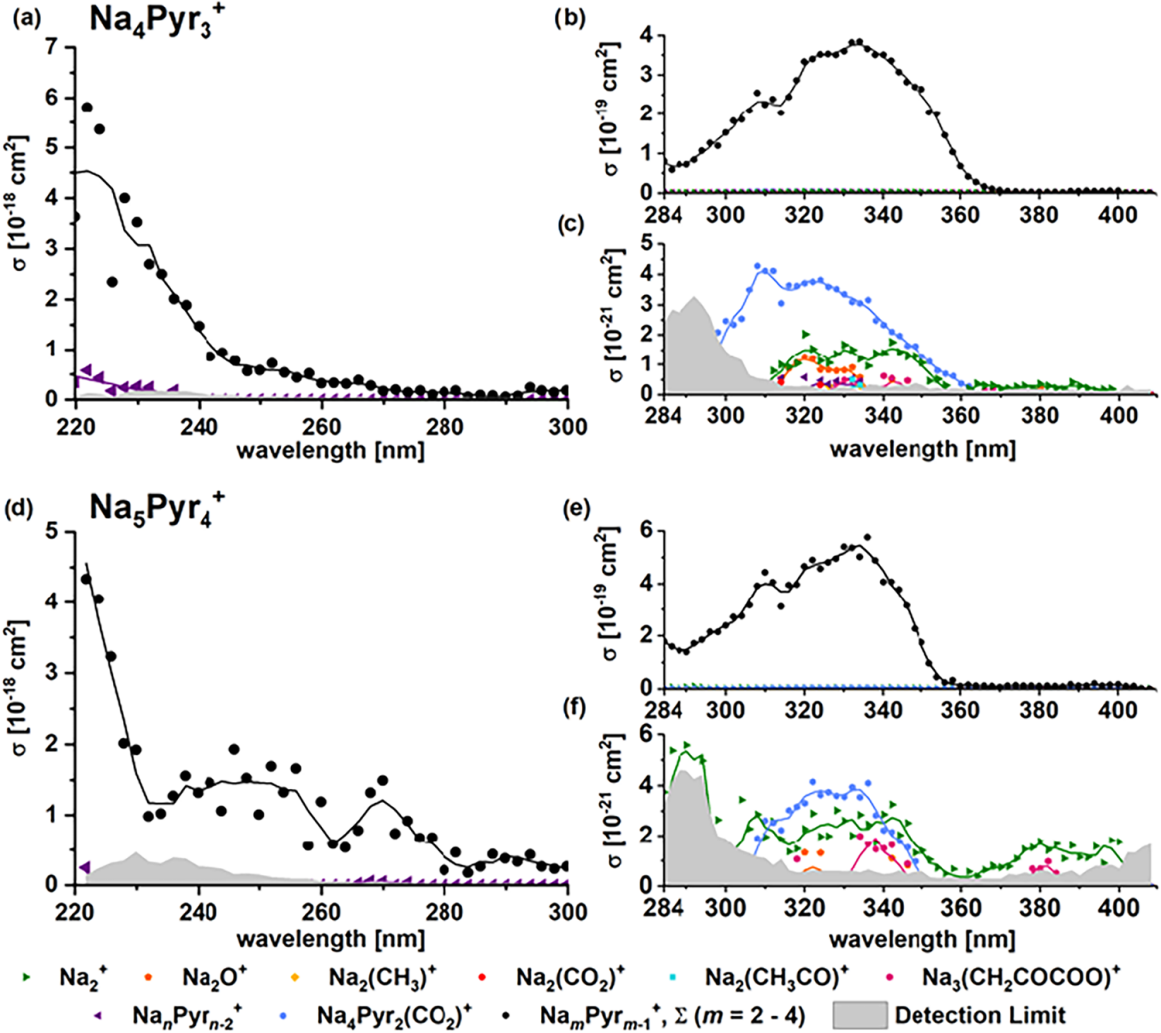
Fragment-resolved UV photodissociation spectra of Na_*n*_Pyr_*n*–1_^+^, *n* = 4,5, with an irradiation time of 2 s in the range of 220–300 nm (a, d) and 20 s in the range of 284–410 nm (b, c and e, f). The lines represent a 6-point running average using the Savitzky–Golay method. The plots shown in (c) and (f) are a zoom-in of the *y*-axis of the data displayed in (b) and (e), respectively.

Figure 2 shows the calculated absorption spectra for Na_*n*_Pyr_*n*–1_^+^, *n* = 4,5. Excited state calculations were performed for the three lowest-lying isomers of both cluster sizes, as depicted in Figure 3. Empirically broadened absorption spectra were modeled by employing an empirical Gaussian broadening of 0.3 eV for all transitions. Calculated structures and energetics of potential fragments are summarized in Figure 4.

**Figure 2:**
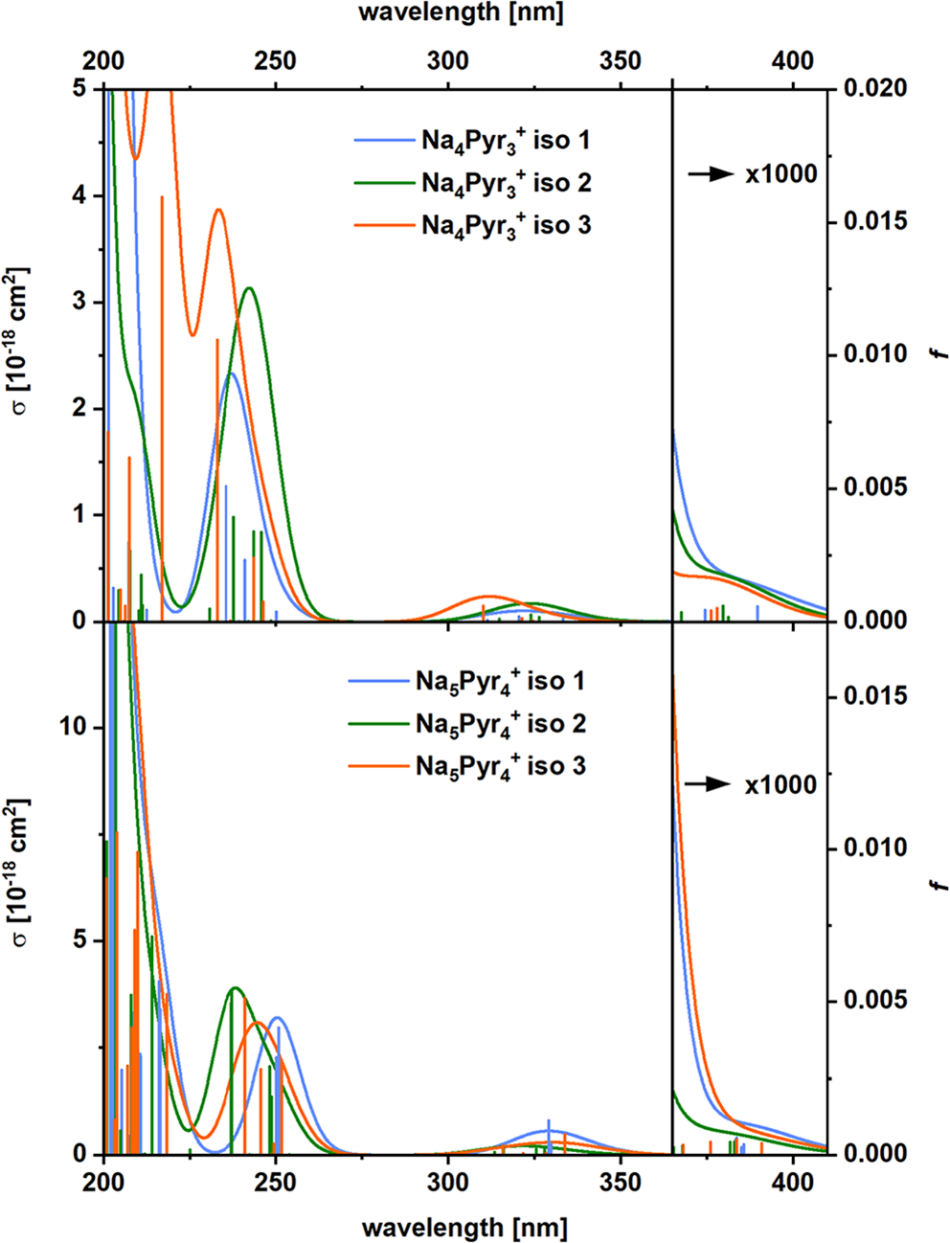
Calculated spectra of Na_4_Pyr_3_^+^ and Na_5_Pyr_4_^+^ at the ωBX97-D/def2-TZVPPD level of theory. The bars show the positions and oscillator strengths of the transitions, and the lines depict the absorption cross sections. The width employed for the spectra was 0.3 eV. Data above 365 nm is multiplied by a factor of 1000 for better visibility of the weak triplet excitations around 380 nm.

**Figure 3:**
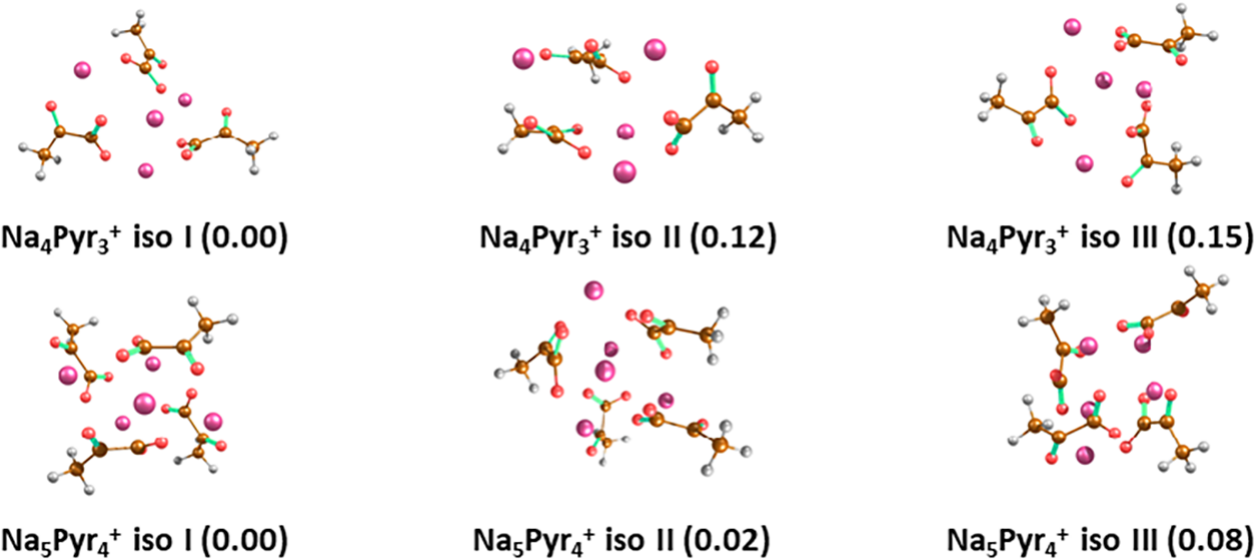
Structures and relative energies (in eV) of the precursor ions Na_4_Pyr_3_^+^ and Na_5_Pyr_4_^+^ calculated at the ωB97XD/aug-cc-pVDZ level of theory. All energies are zero-point-corrected. All structures have a singlet spin state, *i.e*., the ground state is S_0_. Color code: Na, pink; C, brown; O, red; H, white.

**Figure 4:**
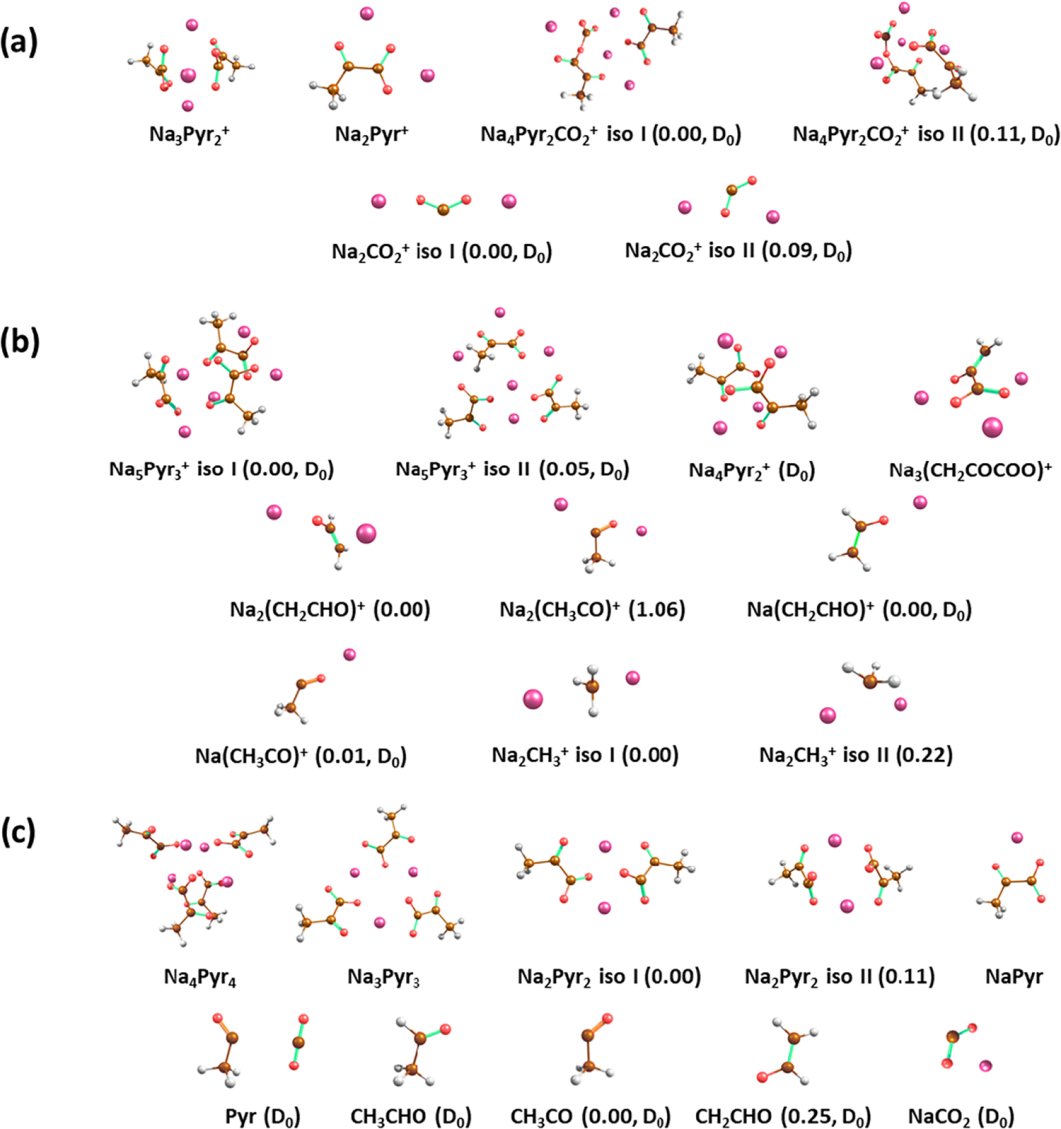
Structures and relative energies (in eV) of (a) primary fragments, (b) further ionic fragments, and (c) selected neutral fragments calculated at the ωB97XD/aug-cc-pVDZ level of theory. All energies are zero-point-corrected. Radicals are labeled with *D*_0_, and all other species have S_0_ ground states. Color code: Na, pink; C, brown; O, red; H, white.

Several studies suggest that not only are singlet excited states of pyruvic acid or pyruvate important for its photolysis, but also the triplet states can play a vital role.^18,19,74,75^ Verlet and co-workers determined the adiabatic singlet-triplet gap to be 0.29 ± 0.04 eV, suggesting potential triplet absorptions toward 380 nm for the systems investigated here. This could explain the absorption at longer wavelengths, as shown in Figure 1. Therefore, the calculations shown in Figure 2 also include triplet excited states.

Comparing the calculated and the experimental spectra shown in Figure 1, the onset of the absorptions, as well as the positions of the bands and the magnitude of the photodissociation cross sections, agree well with the experimental findings. It is apparent from these computational results that it is not possible to clearly assign one dominant isomer in the experiment on the basis of the photodissociation spectra. Most likely, several isomers contribute to the absorption bands, as the spectra were measured at room temperature, and the three lowest-lying isomers were within 0.15 eV. In the actinic region, three and four (*n*,π*) transitions from the S_0_ to the S_1_ state were observed for Na_4_Pyr_3_^+^ and Na_5_Pyr_4_^+^, respectively, corresponding to the number of pyruvate ions in the cluster. The band positions depend on the interaction of Pyr^–^ with Na^+^ and other Pyr^–^ in the cluster, which explains the broad absorption observed in the experiment. The absorptions tentatively assigned to the triplet states lie lower in energy than the singlet excitations, as anticipated, and their position as well as intensity are comparable to the weak absorption above ≈380 nm, as shown in Figure 1.

Fragmentation proceeds mostly by the loss of neutral stoichiometric sodium pyruvate units (Na_*m*_Pyr_*m*_), resulting in Na_*n–m*_Pyr_*n*–*m*–1_^+^ fragments detected in the mass spectra (Figure 1b,e and reactions 1–5 in Table 1). For better visibility, the total photodissociation cross section is not displayed there, as it is almost identical to the partial cross section of the stoichiometric fragments. Nonstoichiometric fragments are observed with 2 orders of magnitude smaller partial cross sections, as displayed in the enlarged sections of the spectra in Figure 1c,f. The most prominent nonstoichiometric fragment is Na_*n*–*m*_Pyr_*n*–*m*–2_CO_2_^+^ (reaction 6 in Table 1), which indicates C–C bond photolysis, followed by Na_*n*–*m*_Pyr_*n*–*m*–2_^+^, formally resulting from the charge transfer and elimination of neutral Pyr (reaction 7). Other fragments were observed in traces, namely Na_2_CH_3_^+^, Na_2_CH_3_CO^+^, and Na_3_CH_2_COCOO^+^ (reactions 10–12). We note that the Na_2_^+^ fragment is observed at wavelengths up to 400 nm, albeit barely above the detection limit, while C–C photolysis stops around 360 nm. However, there is some residual Na_2_^+^ ion signal even without laser irradiation; thus, the Na_2_^+^ signal above 360 nm might be due to imperfect background correction.

**Table 1:** Energies Δ*E*_0_ (in eV) of Potential Primary Photodissociation Channels Calculated at the ωB97XD/aug-cc-pVDZ Level^a^

Reaction	**Δ** ***E***_**0**_ **/eV**		
**Na**_***n***_ **Pyr**_***n***–**1**_^**+**^ **+** ***hν*** **→**	***n*** **= 2**	***n*** **= 4**	***n*** **= 5**
${\mathrm{Na}}^{+}+{\mathrm{Na}}_{n-1}{\mathrm{Pyr}}_{n-1}$ 2	2.36	2.55	2.52
3 ${\mathrm{Na}}_{n-1}{{\mathrm{Pyr}}_{n-2}}^{+}+\mathrm{NaPyr}$		2.18	2.21
4 ${\mathrm{Na}}_{n-2}{{\mathrm{Pyr}}_{n-3}}^{+}+{\mathrm{Na}}_{2}{\mathrm{Pyr}}_{2}$		2.06	2.13
5 ${\mathrm{Na}}_{n-3}{{\mathrm{Pyr}}_{n-4}}^{+}+{\mathrm{Na}}_{3}{\mathrm{Pyr}}_{3}$			2.41
6 ${\mathrm{Na}}_{n-m}{\mathrm{Pyr}}_{n-m-2}{{\mathrm{CO}}_{2}}^{+}+{\mathrm{Na}}_{m}{\mathrm{Pyr}}_{m}+{\mathrm{CH}}_{3}\mathrm{CO}$	*m* = 0: 3.80	*m* = 0: 3.34	*m* = 1: *5.55*
		*m* = 2: *5.86*	
7a ${\mathrm{Na}}_{n-m}{{\mathrm{Pyr}}_{n-m-2}}^{+}+{\mathrm{Na}}_{m}{\mathrm{Pyr}}_{m}+\mathrm{Pyr}$	*m* = 0: 4.81	*m* = 0: 3.67	*m* = 0: 3.79
		*m* = 2: *6.88*	*m* = 1: *5.88*
			*m* = 3: *7.22*
7b ${\mathrm{Na}}_{n-m}{{\mathrm{Pyr}}_{n-m-2}}^{+}+{\mathrm{Na}}_{m}{\mathrm{Pyr}}_{m}+{\mathrm{CH}}_{3}\mathrm{CO}+{\mathrm{CO}}_{2}$	*m* = 0: 4.87	*m* = 0: 3.73	*m* = 0: 3.85
		*m* = 2: *6.94*	*m* = 1: *5.94*
			*m* = 3: *7.28*
7c ${\mathrm{Na}}_{n-m}{{\mathrm{Pyr}}_{n-m-2}}^{+}+{\mathrm{Na}}_{m}{\mathrm{Pyr}}_{m}+{\mathrm{CH}}_{2}\mathrm{COCOOH}$	*m* = 0: 5.31	*m* = 0: 4.16	*m* = 0: 4.29
		*m* = 2: *7.37*	*m* = 1: *6.37*
			*m* = 3: *7.71*
8 ${\mathrm{Na}}_{2}{{\mathrm{CH}}_{3}}^{+}+{\mathrm{Na}}_{n-2}{\mathrm{Pyr}}_{n-2}+{\mathrm{CO}}_{2}+\mathrm{CO}$	3.25	*5.31*	
9 ${\mathrm{Na}}_{2}{\mathrm{CH}}_{2}{\mathrm{CHO}}^{+}+{\mathrm{Na}}_{n-2}{\mathrm{Pyr}}_{n-2}+{\mathrm{CO}}_{2}$	1.45	3.51	
10a ${\mathrm{Na}}_{3}{\left({\mathrm{CH}}_{2}\mathrm{COCOO}\right)}^{+}+{\mathrm{Na}}_{n-3}{\mathrm{Pyr}}_{n-3}+{\mathrm{CH}}_{3}\mathrm{CO}+{\mathrm{HCO}}_{2}$		*8.94*	*8.89*
10b ${\mathrm{Na}}_{3}{\left({\mathrm{CH}}_{2}\mathrm{COCOO}\right)}^{+}+{\mathrm{Na}}_{n-3}{\mathrm{Pyr}}_{n-3}+{\mathrm{CH}}_{3}\mathrm{COCOOH}$		4.95	4.89
10c ${\mathrm{Na}}_{3}{\left({\mathrm{CH}}_{2}\mathrm{COCOO}\right)}^{+}+{\mathrm{Na}}_{n-3}{\mathrm{Pyr}}_{n-3}+{\mathrm{CH}}_{3}\mathrm{CHO}+{\mathrm{CO}}_{2}$		4.75	4.69
11a ${\mathrm{NaCH}}_{3}{\mathrm{CO}}^{+}+{\mathrm{NaCO}}_{2}$	*4.61*		
11b ${\mathrm{NaCH}}_{3}{\mathrm{CO}}^{+}+\mathrm{Na}+{\mathrm{CO}}_{2}$	*4.84*		

a Values in italics exceed the available photon energy.

Some fragments containing only two Na^+^ ions, such as Na_2_CH_3_^+^ and Na_2_O^+^, may be secondary products formed by the photolysis of the stoichiometric Na_2_Pyr^+^ fragment. Considering the 20 s irradiation time, which corresponds to 400 laser pulses at a 20 Hz pulse repetition rate, this seems entirely plausible. We therefore recorded a photodissociation spectrum of Na_2_Pyr^+^ in the 305–370 nm range, as shown in Figure 5. Unfortunately, the lower limit of the mass spectrometer is *m*/*z* 28.8, so the Na^+^ fragment ion at *m*/*z* 22.990 cannot be detected. Nevertheless, the photofragments shown in Figure 5 provide important information on pyruvate photochemistry in the presence of sodium ions. Indeed, Na_2_CO_2_^+^ and, to a lesser extent, Na_2_CH_3_CO^+^ are observed, both of which require C–C bond photolysis for their formation (reactions 6 and 11). Interestingly, NaCH_3_CO^+^ is also present close to the detection limit, implying the loss of neutral NaCO_2_ or Na + CO_2_ (reactions 15 and 16). Na_2_CH_3_^+^ is probably formed from Na_2_CH_3_CO^+^ by the elimination of CO (reaction 17 in Table 2). The Na_2_CO_2_^+^ fragment is a plausible precursor for Na_2_O^+^ and Na_2_^+^ as secondary fragments (reactions 18 and 19), which actually dominate the spectrum after irradiating for 20 s.

**Figure 5:**
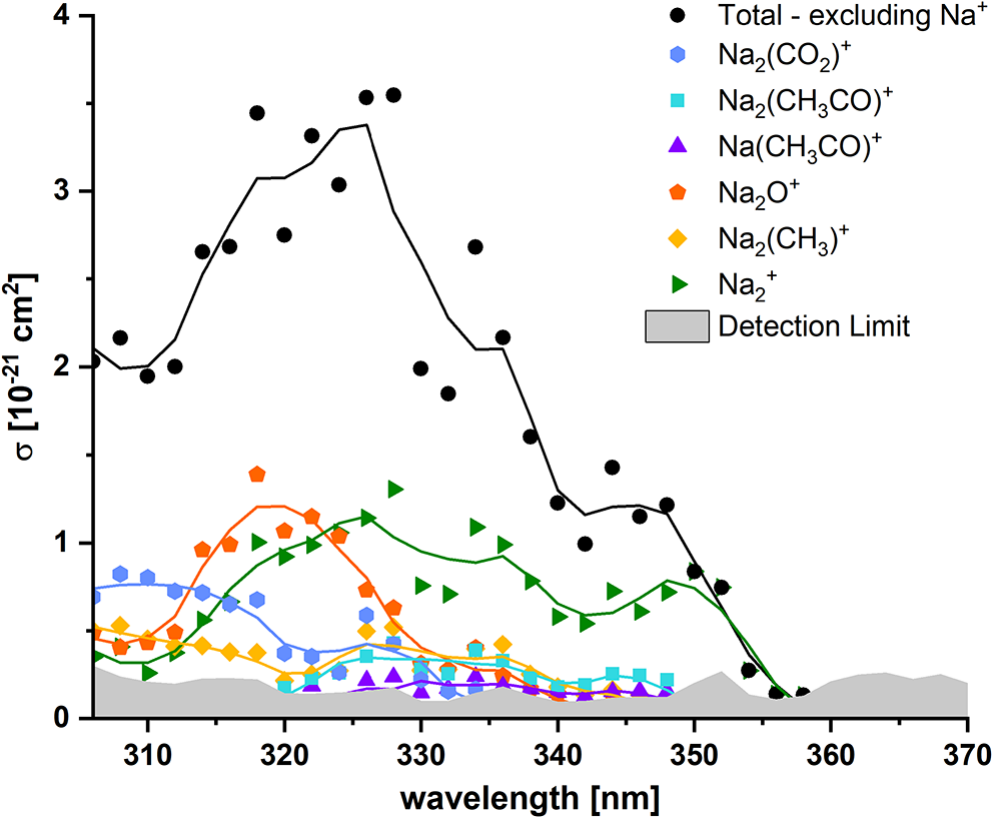
Fragment-resolved UV photodissociation spectrum of the smallest cluster size Na_2_Pyr^+^ measured with an irradiation time of 20 s. The lines represent 6-point running averages using the Savitzky-Golay method. The stoichiometric fragment Na^+^ lies below the lower mass limit of the instrument of *m*/*z* = 28.8 u and therefore cannot be detected.

**Table 2:** Energies Δ*E*_0_ of Potential Secondary Photodissociation Channels and Other Relevant Dissociation Reactions Calculated at the ωB97XD/aug-cc-pVDZ Level

**reaction**	**Δ** ***E***_**0**_ **/eV**
11 ${\mathrm{Na}}_{2}{\mathrm{CH}}_{2}{\mathrm{CHO}}^{+}\to {\mathrm{Na}}_{2}{{\mathrm{CH}}_{3}}^{+}+\mathrm{CO}$	1.80
12 ${\mathrm{Na}}_{2}{{\mathrm{CO}}_{2}}^{+}\to {\mathrm{Na}}_{2}O^{+}+\mathrm{CO}$	2.30
13 ${\mathrm{Na}}_{2}{{\mathrm{CO}}_{2}}^{+}\to {{\mathrm{Na}}_{2}}^{+}+{\mathrm{CO}}_{2}$	1.07
14 ${\mathrm{Na}}_{4}{\mathrm{Pyr}}_{2}{{\mathrm{CO}}_{2}}^{+}\to {\mathrm{Na}}_{4}{{\mathrm{Pyr}}_{2}}^{+}+{\mathrm{CO}}_{2}$	0.38
15 ${\mathrm{Na}}_{2}{\mathrm{CH}}_{2}{\mathrm{CHO}}^{+}\to {\mathrm{NaCH}}_{3}{\mathrm{CO}}^{+}+\mathrm{Na}$	3.37
16 ${\mathrm{Na}}_{4}{\mathrm{Pyr}}_{2}{{\mathrm{CO}}_{2}}^{+}\to {\mathrm{Na}}_{3}{{\mathrm{Pyr}}_{2}}^{+}+{\mathrm{NaCO}}_{2}$	2.08
17 ${\mathrm{Na}}_{4}{{\mathrm{Pyr}}_{2}}^{+}\to {\mathrm{Na}}_{3}{{\mathrm{Pyr}}_{2}}^{+}+\mathrm{Na}$	1.92
18 ${\mathrm{Na}}_{4}{{\mathrm{Pyr}}_{2}}^{+}\to {\mathrm{Na}}^{+}+{\mathrm{Na}}_{3}{\mathrm{Pyr}}_{2}$	2.64
19a ${\mathrm{Na}}_{3}{{\mathrm{Pyr}}_{2}}^{+}\to {\mathrm{Na}}_{3}{\left({\mathrm{CH}}_{2}\mathrm{COCOO}\right)}^{+}+{\mathrm{CH}}_{3}\mathrm{COCOOH}$	2.77
19b ${\mathrm{Na}}_{3}{{\mathrm{Pyr}}_{2}}^{+}\to {\mathrm{Na}}_{3}{\left({\mathrm{CH}}_{2}\mathrm{COCOO}\right)}^{+}+{\mathrm{CH}}_{3}\mathrm{CHO}+{\mathrm{CO}}_{2}$	2.57
20 ${\mathrm{Na}}_{2}{{\mathrm{CO}}_{2}}^{+}\to {\mathrm{Na}}^{+}+{\mathrm{NaCO}}_{2}$	1.79
21 ${\mathrm{Na}}_{2}{\mathrm{CH}}_{2}{\mathrm{CHO}}^{+}\to {\mathrm{Na}}^{+}+{\mathrm{NaCH}}_{3}\mathrm{CO}$	3.01
22 ${\mathrm{Na}}_{2}{\mathrm{CH}}_{2}{\mathrm{CHO}}^{+}\to {{\mathrm{Na}}_{2}}^{+}+{\mathrm{CH}}_{3}\mathrm{CO}$	3.43
23 ${\mathrm{Na}}_{2}{{\mathrm{CH}}_{3}}^{+}\to {{\mathrm{Na}}_{2}}^{+}+{\mathrm{CH}}_{3}$	2.30
24 ${\mathrm{Na}}_{2}{{\mathrm{CH}}_{3}}^{+}\to {\mathrm{Na}}^{+}+{\mathrm{NaCH}}_{3}$	1.84
25 ${{\mathrm{Na}}_{2}}^{+}\to {\mathrm{Na}}^{+}+\mathrm{Na}$	0.95

To further test the hypotheses on the precursors of the various fragments, we recorded photodissociation kinetics of Na_4_Pyr_3_^+^ and Na_2_Pyr^+^ at 345 and 318 nm, respectively (see SI, Figures S1 and S2 and Tables S1 and S2). Both fits are consistent with the direct elimination of neutral Pyr (reaction 7) to yield the charge-transfer fragments Na_4_Pyr_2_^+^ and Na_2_^+^ as primary products. However, the Na_4_Pyr_2_CO_2_^+^ fragment predominantly eliminates CO_2_ and thus also contributes to the intensity of the Na_4_Pyr_2_^+^ charge-transfer fragment (reaction 20). The Na_2_Pyr^+^ kinetics (Figure S2) clearly identifies Na_2_CO_2_^+^ as a precursor of Na_2_O^+^ (reaction 18), which does not seem to fragment further at 318 nm. Both fits indicate an incomplete overlap of the ion cloud with the laser beam at 50% in the experiments. If the overlap was the same in the experiments shown in Figures 1 and 5, the actual cross sections are about twice as high as those shown in the figures.

The secondary photodissociations derived from the kinetic fit in Figure S2 and Table S2 can only occur if the fragments have absorptions at the given laser wavelengths. We therefore simulated the absorption spectra of Na_2_Pyr^+^, Na_2_CH_2_CHO^+^, Na_2_CO_2_^+^, Na_2_CH_3_^+^, Na_2_O^+^, and Na_2_^+^, as displayed in Figure 6. All of them show intense absorption behavior in the lower wavelength region, starting below 300 nm, except for Na_2_^+^. However, only three of them, namely Na_2_Pyr^+^, Na_2_CO_2_^+^, and Na_2_CH_3_^+^, exhibit absorption bands in the 300–400 nm range. This is consistent with the secondary fragmentation of Na_2_CO_2_^+^ and Na_2_CH_3_^+^ in the laser kinetics shown in Figure S2. It is important to note that the fit for the laser kinetics shown in Figure S2 and Table S2 is not unique, as the fragment intensities are in the subpercentage regime. However, the fit presented here is consistent with all the other findings.

**Figure 6:**
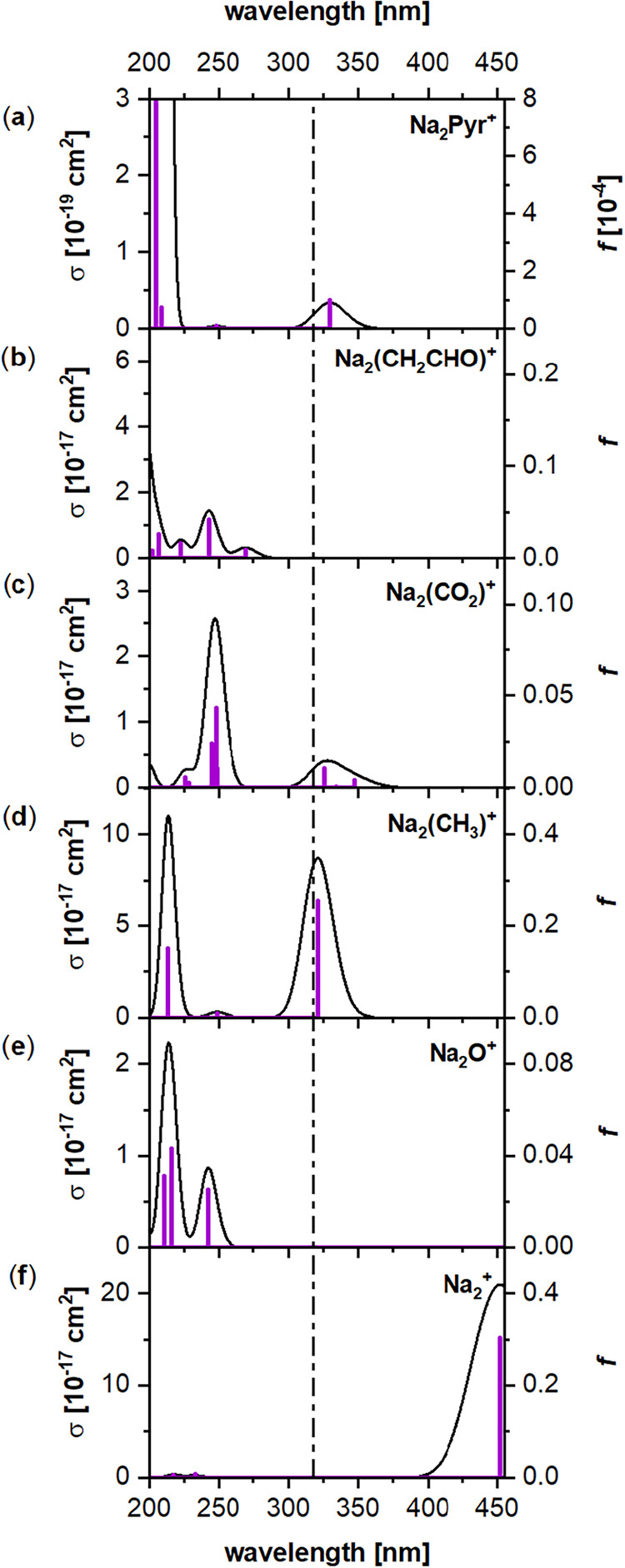
Calculated absorption cross sections and oscillator strengths for (a) Na_2_Pyr^+^, (b) Na_2_CH_2_CHO^+^, (c) Na_2_(CO_2_)^+^, (d) Na_2_(CH_3_)^+^, (e) Na_2_O^+^, and (f) Na_2_^+^ at the ωBX97-D/def2-TZVPPD level of theory. The bars show the positions and oscillator strengths of the transitions, and the lines depict the absorption cross sections. The width employed for the spectra was 0.3 eV. A dash-dotted line at 318 nm marks the wavelength, where the kinetics were recorded (see Figure S2).

Obviously, the observed dissociation is caused by the absorption of a photon. For a mechanistic understanding, however, it is important to test whether photodissociation leads to products that are qualitatively different from activation methods that work without electronic excitation. We therefore performed SORI-CID on Na_*n*_Pyr_*n*–1_^+^, *n* = 1–4. Here, the cluster size Na_3_Pyr_2_^+^ was added, as the signal-to-noise ratio was sufficient for SORI-CID experiments, but not enough to record a photodissociation spectrum. Figure S3 shows the fragment intensities as a function of the SORI power. In each case, nonstoichiometric fragments are observed in abundances comparable to those in the photodissociation experiments. At high SORI powers, even the weakest photodissociation products, such as Na­(CH_3_CO)^+^ and Na_2_(CH_3_CO)^+^, are present in very limited amounts. This implies that either the relevant electronically excited states can be reached from the ground state via conical intersections, or fragmentation proceeds statistically in the electronic ground state, in the case of photoexcitation after internal conversion, as these are the possibilities available for gas-phase photochemistry. We have drawn similar conclusions before for the glyoxylate case, based on a computational analysis of the excited state potential energy surface along the C–C bond dissociation coordinate.^6^ Miller and Uggerud reported C–C bond cleavage of gas-phase pyruvate as the major fragmentation pathway in CID experiments,^58^ in the absence of the energetically lower-lying stoichiometric fragmentation pathways available in sodium pyruvate clusters.

To better understand the origin of the nonstoichiometric fragments, we calculated their structures and the energetics of the potential fragmentation pathways (see Figure 4 and Tables 1 and 2, reactions 6–32). The actinic absorption band stretches from ≈290–360 nm, corresponding to a photon energy of 3.4–4.3 eV. In this band, the dominant nonstoichiometric fragmentation pathway is C–C bond photolysis, with CO_2_^–^ remaining in the cluster (reaction 6 in Table 1). For Na_4_Pyr_3_^+^, the calculated energy of 3.34 eV is available throughout the band. For Na_5_Pyr_4_^+^, the observation that NaPyr is always lost in addition to CH_3_CO indicates that Na_4_Pyr_2_CO_2_^+^ is a secondary fragment, as the total required energy of 5.55 eV is not available with a single photon.

The most stable structures of the C–C bond photolysis product Na_4_Pyr_2_(CO_2_)^+^ found in our extensive structure search feature a quite unusual CH_3_COC­(O)­OCO_2_^2–^ radical dianion (Figures S4 and S5), with the spin density localized mostly in a π-type orbital along the central 1,2-dicarbonyl unit. The total natural charge on this dianion is −1.92, with −0.65 on the terminal CO_2_ subunit (see Table S3). Not unexpectedly, this species is easily decarboxylated, with an energy of 0.38 eV (reaction 20).

This leads to the formal charge-transfer products Na_4_Pyr_2_^+^ and Na_5_Pyr_3_^+^, which are the predominant nonstoichiometric fragments below 300 nm. Elimination of neutral Pyr, i.e., the CH_3_COCOO radical, is highly unlikely, as decomposition to CH_3_CO + CO_2_ requires only 0.06 eV (reactions 7 and 8). The keto–enol tautomeric rearrangement of Pyr to CH_2_COCOOH (reaction 9) requires significant extra energy and is thus also ruled out. The formal charge transfer is therefore interpreted as C–C bond photolysis, followed by the loss of CH_3_CO and CO_2_ from the cluster. This is in line with the excited state calculations, which do not show any indication of a charge-transfer transition. Natural population analysis of Na_4_Pyr_2_^+^ and Na_5_Pyr_3_^+^ reveals that the extra electron available after the elimination of neutral CH_3_CO + CO_2_ is localized on one Pyr unit, with a total natural charge of −1.90, compared to −0.96 and −0.94 for the other Pyr units in Na_4_Pyr_2_^+^ and Na_5_Pyr_3_^+^, respectively, Table S3. A plot of the spin density (see Figures S6 and S7) reveals that this electron is mostly localized in the π* orbital of the keto group, which is also reflected in the natural charges on these atoms. The radical dianion CH_3_COCOO^2–^, which is formed by C–C bond photolysis followed by CO_2_ elimination from the cluster, has repeatedly been reported in pulse radiolysis^76,77^ and EPR spectroscopy,^78–81^ but has not received much attention recently. All other nonstoichiometric fragments of Na_4_Pyr_3_^+^ and Na_5_Pyr_4_^+^ are clearly secondary fragments, as the required energy is well above the available photon energy.

C–C bond photolysis in Na_2_Pyr^+^ leads to Na_2_CH_3_CO^+^ at longer wavelengths, which requires only 1.44 eV (reaction 11), in contrast to the 3.80 eV required for Na_2_CO_2_^+^ (reaction 6). Interestingly, the latter becomes the dominant fragment when sufficient photon energy is available. CO loss from Na_2_CH_3_CO^+^ is also possible with the available photon energy, consistent with the observation of Na_2_CH_3_^+^ (reaction 10). NaCH_3_CO^+^ may originate from Na_2_CH_3_CO^+^ via the loss of Na (reaction 21), as the direct formation (reactions 15 and 16) is energetically inaccessible. In the 310–330 nm region, Na_2_O^+^ is prominent, which on energetic grounds alone is a secondary product. The kinetics fit, Figure S2, is consistent with the formation of Na_2_O^+^ from Na_2_CO_2_^+^, and the required energy of 2.30 eV (reaction 18) is below the photon energy. This is a rare case of C–O bond photolysis in a CO_2_^–^ moiety, previously reported by Sanov for hydrated CO_2_^–^.^82^ Reactions 22–32 in Table 2 summarize the reactions that likely occur to a minor extent in the sequential photodissociation of sodium pyruvate clusters.

Verlet and co-workers, as well as Wang and co-workers, observed C–C bond photolysis in gas-phase Pyr^–^, resulting in CH_3_CO^–^ + CO_2_, which amounts to heterolytic bond cleavage, with the electron pair of the C–C bond turning into a lone pair at the carbonyl-C atom. Subsequent CO elimination leads to the formation of CH_3_^–^. This behavior is mirrored in the Na_2_CH_3_CO^+^ and Na_2_CH_3_^+^ products of Na_2_Pyr^+^. In larger sodium pyruvate clusters, however, C–C photolysis proceeds via heterolytic cleavage, with the formal CO_2_^–^ fragment interacting with a second Pyr^–^ unit, ultimately leading to CO_2_ elimination and the Pyr^2–^ radical dianion. This formal charge transfer does not result from a charge-transfer excitation, but is an indirect result of C–C bond photolysis.

## Conclusions

The UV/vis photodissociation experiments of Na_*n*_Pyr_*n*–1_^+^, *n* = 2, 4, 5, reveal rich photochemistry in the actinic region. Quantum chemical calculations suggest that the broad transitions measured for larger cluster sizes stem from the contribution of several isomers, with the shift in the position of the (*n*,π*) transition of the pyruvate units depending on their detailed interaction with the cluster environment. Besides the main fragmentation pathway toward stoichiometric fragments, formation of nonstoichiometric fragments starts with C–C bond photolysis of a pyruvate chromophore. In larger clusters, a CH_3_CO radical is lost initially, and the subsequent elimination of CO_2_ completes a formal charge transfer from one pyruvate anion to another in the cluster, which amounts to a quite unusual charge transfer between two anions forming a radical dianion, made possible by the stabilizing interaction with the cations in the salt cluster. This process provides a CH_3_CO and CH_3_COCOO^2–^ radical pair, which can initiate and sustain the complex reaction cascade observed in photochemical studies of sodium pyruvate. In the smallest clusters containing only one pyruvate molecular anion, CO_2_^–^ or CH_3_^–^ units formed by C–C bond photolysis are stabilized by two Na^+^ ions.

## Supplementary Material





## Notes

Additional experimental results on laser kinetics and SORI-CID spectra; theoretical calculations of the spin densities and natural charges for Na_4_Pyr_2_(CO_2_)^+^, Na_4_Pyr_2_^+^, and Na_5_Pyr_3_^+^, as well as optimized geometries for all isomers (PDF)
Numerical data shown in Figures 1, 2, 5, 6, S1, S2, S3 XLSX

## References

[1] Finlayson-Pitts, B. J. ; Pitts, J. N. Chemistry of the Upper and Lower Atmosphere. Theory, Experiments, and Applications; Academic Press: San Diego, Calif., 2000.

[2] Moortgat G. K. (2001). Important
photochemical processes in the atmosphere. Pure
Appl. Chem..

[3] Epstein S. A., Nizkorodov S. A. (2012). A comparison of the chemical sinks of atmospheric organics
in the gas and aqueous phase. Atmos. Chem. Phys..

[4] Reed
Harris A. E., Cazaunau M., Gratien A., Pangui E., Doussin J.-F., Vaida V. (2017). Atmospheric Simulation Chamber Studies
of the Gas-Phase Photolysis of Pyruvic Acid. J. Phys. Chem. A.

[5] Renard P., Reed Harris A. E., Rapf R. J., Ravier S., Demelas C., Coulomb B., Quivet E., Vaida V., Monod A. (2014). Aqueous Phase
Oligomerization of Methyl Vinyl Ketone by Atmospheric Radical Reactions. J. Phys. Chem. C.

[6] Bersenkowitsch N. K., Ončák M., van der Linde C., Herburger A., Beyer M. K. (2018). Photochemistry of glyoxylate embedded in sodium chloride
clusters, a laboratory model for tropospheric sea-salt aerosols. Phys. Chem. Chem. Phys..

[7] Hofzumahaus A., Kraus A., Kylling A., Zerefos C. S. (2002). Solar actinic radiation
(280–420 nm) in the cloud-free troposphere between ground and
12 km altitude: Measurements and model results. J. Geophys. Res.:Atmos..

[8] Vesley G. F., Leermakers P. A. (1964). The Photochemistry of α-Keto Acids and α-Keto
Esters. III. Photolysis of Pyruvic Acid in the Vapor Phase. J. Phys. Chem. A.

[9] Yamamoto S., Back R. A. (1985). The photolysis and thermal decomposition of pyruvic
acid in the gas phase. Can. J. Chem..

[10] Horowitz A., Meller R., Moortgat G. K. (2001). The UV–VIS
absorption cross
sections of the α-dicarbonyl compounds: pyruvic acid, biacetyl
and glyoxal. J. Photoch. Photobio. A.

[11] Mellouki A., Mu Y. (2003). On the atmospheric
degradation of pyruvic acid in the gas phase. J. Photoch. Photobio. A.

[12] O’Neill J. A., Kreutz T. G., Flynn G. W. (1987). IR diode laser study
of vibrational
energy distribution in CO_2_ produced by UV excimer laser
photofragmentation of pyruvic acid. J. Chem.
Phys..

[13] Rosenfeld R. N., Weiner B. (1983). Energy disposal
in the photofragmentation of pyruvic
acid in the gas phase. J. Am. Chem. Soc..

[14] Wood C. F., O’Neill J. A., Flynn G. W. (1984). Infrared diode laser probes of photofragmentation
products: Bending excitation in CO_2_ produced by excimer
laser photolysis of pyruvic acid. Chem. Phys.
Lett..

[15] Samanta B. R., Fernando R., Rösch D., Reisler H., Osborn D. L. (2020). Looking
at the bigger picture: Identifying the photoproducts of pyruvic acid
at 193 nm. J. Chem. Phys..

[16] Sutradhar S., Samanta B. R., Fernando R., Reisler H. (2019). Spectroscopy and Two-Photon
Dissociation of Jet-Cooled Pyruvic Acid. J.
Phys. Chem. A.

[17] Dhanya S., Maity D. K., Upadhyaya H. P., Kumar A., Naik P. D., Saini R. D. (2003). Dynamics of OH formation
in photodissociation of pyruvic
acid at 193 nm. J. Chem. Phys..

[18] Sauer L. J., Davis H. F. (2025). Unraveling the Primary
Photochemistry of Pyruvic Acid:
Direct Observation of Three Competing Channels. J. Phys. Chem. Lett..

[19] Burrow E. M., Carmona-García J., Clarke C. J., Curchod B. F. E., Verlet J. R. R. (2025). The Singlet-Triplet Gap of Pyruvic
Acid. J. Am. Chem. Soc..

[20] Jarraya M., Bellili A., Barreau L., Cubaynes D., Garcia G. A., Poisson L., Hochlaf M. (2022). Probing the
dynamics of the photo-induced
decarboxylation of neutral and ionic pyruvic acid. Faraday Discuss..

[21] da
Silva G. (2016). Decomposition of Pyruvic Acid on the Ground-State Potential Energy
Surface. J. Phys. Chem. A.

[22] Yang D., Zhang L. (2012). Excited-state hydrogen
bonding dynamics of pyruvic acid and geminal-diol,
2,2-dihydroxypropanoic acid in aqueous solution: a DFT/TDDFT study. J. Phys. Org. Chem..

[23] Chang X.-P., Fang Q., Cui G. (2014). Mechanistic photodecarboxylation
of pyruvic acid: excited-state proton transfer and three-state intersection. J. Chem. Phys..

[24] Valadbeigi Y., Farrokhpour H. (2013). Theoretical
study on keto–enol tautomerism and
isomerization in pyruvic acid. Int. J. Quantum
Chem..

[25] Hutton L., Curchod B. F. E. (2022). Photodynamics
of Gas-Phase Pyruvic Acid Following Light
Absorption in the Actinic Region. ChemPhotoChem.

[26] Murto J., Raaska T., Kunttu H., Räsänen M. (1989). Conformers
and vibrational spectra of pyruvic acid: an ab initio study. J. Mol. Struct.:THEOCHEM.

[27] Church J. R., Vaida V., Skodje R. T. (2021). Kinetic
Study of Gas-Phase Reactions
of Pyruvic Acid with HO_2_. J. Phys.
Chem. A.

[28] Schnitzler E. G., Seifert N. A., Ghosh S., Thomas J., Xu Y., Jäger W. (2017). Hydration of the simplest α-keto acid: a rotational
spectroscopic and ab initio study of the pyruvic acid-water complex. Phys. Chem. Chem. Phys..

[29] Grosjean D. (1983). Atmospheric
reactions of pyruvic acid. Atmos. Environ..

[30] Shemesh D., Luo M., Grassian V. H., Gerber R. B. (2020). Absorption spectra of pyruvic acid
in water: insights from calculations for small hydrates and comparison
to experiment. Phys. Chem. Chem. Phys..

[31] Luo M., Shemesh D., Sullivan M. N., Alves M. R., Song M., Gerber R. B., Grassian V. H. (2020). Impact
of pH and NaCl and CaCl_2_ Salts on the Speciation and Photochemistry
of Pyruvic Acid
in the Aqueous Phase. J. Phys. Chem. A.

[32] Eugene A. J., Guzman M. I. (2017). Reactivity of Ketyl
and Acetyl Radicals from Direct
Solar Actinic Photolysis of Aqueous Pyruvic Acid. J. Phys. Chem. A.

[33] Closs G. L., Miller R. J. (1978). Photoreduction and photodecarboxylation
of pyruvic
acid. Applications of CIDNP to mechanistic photochemistry. J. Am. Chem. Soc..

[34] Lewis J. S., Gaunt A. P., Comment A. (2022). Photochemistry of pyruvic acid is
governed by photo-induced intermolecular electron transfer through
hydrogen bonds. Chem. Sci..

[35] Guzman M. I., Eugene A. J. (2021). Aqueous Photochemistry
of 2-Oxocarboxylic Acids: Evidence,
Mechanisms, and Atmospheric Impact. Molecules.

[36] Xia S.-S., Eugene A. J., Guzman M. I. (2018). Cross Photoreaction
of Glyoxylic
and Pyruvic Acids in Model Aqueous Aerosol. J. Phys. Chem. A.

[37] Carlton A. G., Turpin B. J., Lim H.-J., Altieri K. E., Seitzinger S. (2006). Link between
isoprene and secondary organic aerosol (SOA): Pyruvic acid oxidation
yields low volatility organic acids in clouds. Geophys. Res. Lett..

[38] Guzmán M. I., Colussi A. J., Hoffmann M. R. (2006). Photoinduced
oligomerization of aqueous
pyruvic acid. J. Phys. Chem. A.

[39] Reed
Harris A. E., Doussin J.-F., Carpenter B. K., Vaida V. (2016). Gas-Phase Photolysis of Pyruvic Acid: The Effect of Pressure on Reaction
Rates and Products. J. Phys. Chem. A.

[40] Blair S. L., Reed Harris A. E., Frandsen B. N., Kjaergaard H. G., Pangui E., Cazaunau M., Doussin J.-F., Vaida V. (2020). Conformer-Specific
Photolysis of Pyruvic Acid and the Effect of Water. J. Phys. Chem. A.

[41] Larsen M. C., Vaida V. (2012). Near infrared photochemistry of pyruvic
acid in aqueous solution. J. Phys. Chem. A.

[42] Griffith E. C., Carpenter B. K., Shoemaker R. K., Vaida V. (2013). Photochemistry of aqueous
pyruvic acid. Proc. Natl. Acad. Sci. U. S. A..

[43] Reed
Harris A. E., Ervens B., Shoemaker R. K., Kroll J. A., Rapf R. J., Griffith E. C., Monod A., Vaida V. (2014). Photochemical kinetics of pyruvic acid in aqueous solution. J. Phys. Chem. A.

[44] Griffith E. C., Shoemaker R. K., Vaida V. (2013). Sunlight-initiated chemistry of aqueous
pyruvic acid: building complexity in the origin of life. Orig. Life Evol. Biosph..

[45] Vaida V. (2005). Sunlight initiated
atmospheric photochemical reactions. Int. J.
Photoenergy.

[46] Rapf R. J., Dooley M. R., Kappes K., Perkins R. J., Vaida V. (2017). pH Dependence
of the Aqueous Photochemistry of α-Keto Acids. J. Phys. Chem. A.

[47] Reed
Harris A. E., Pajunoja A., Cazaunau M., Gratien A., Pangui E., Monod A., Griffith E. C., Virtanen A., Doussin J.-F., Vaida V. (2017). Multiphase Photochemistry of Pyruvic
Acid under Atmospheric Conditions. J. Phys.
Chem. A.

[48] Kappes K. J., Deal A. M., Jespersen M. F., Blair S. L., Doussin J.-F., Cazaunau M., Pangui E., Hopper B. N., Johnson M. S., Vaida V. (2021). Chemistry and Photochemistry of Pyruvic Acid at the Air-Water Interface. J. Phys. Chem. A.

[49] Laskin A., Moffet R. C., Gilles M. K., Fast J. D., Zaveri R. A., Wang B., Nigge P., Shutthanandan J. (2012). Tropospheric
Chemistry of Internally Mixed Sea Salt and Organic Particles: Surprising
Reactivity of NaCl with Weak Organic Acids. J. Geophys. Res..

[50] Su B., Zhuo Z., Fu Y., Sun W., Chen Y., Du X., Yang Y., Wu S., Xie Q., Huang F., Chen D., Li L., Zhang G., Bi X., Zhou Z. (2021). Individual particle investigation on the chloride depletion
of inland
transported sea spray aerosols during East Asian summer monsoon. Sci. Total Environ..

[51] Su B., Wang T., Zhang G., Liang Y., Lv C., Hu Y., Li L., Zhou Z., Wang X., Bi X. (2022). A review of
atmospheric aging of sea spray aerosols: Potential factors affecting
chloride depletion. Atmos. Environ..

[52] Hartmann J. C., Lim J. Y., Sheng Y., Reimann M., Madlener S. J., van der Linde C., Siu C.-K., Beyer M. K. (2026). Salt Cluster With
Surface Defect Shows Anomalous Acid-Base Chemistry. Angew. Chem., Int. Ed..

[53] Beichert P., Finlayson-Pitts B. J. (1996). Knudsen
Cell Studies of the Uptake of Gaseous HNO_3_ and Other Oxides
of Nitrogen on Solid NaCl: The Role of Surface-Adsorbed
Water. J. Phys. Chem. A.

[54] Gard E. E., Kleeman M. J., Gross D. S., Hughes L. S., Allen J. O., Morrical B. D., Fergenson D. P., Dienes T., E G. M., Johnson R. J., Cass G. R., Prather K. A. (1998). Direct Observation
of Heterogeneous Chemistry in the Atmosphere. Science.

[55] Leermakers P. A., Vesley G. F. (1963). The Photochemistry
of α-Keto Acids and α-Keto
Esters. I. Photolysis of Pyruvic Acid and Benzoylformic Acid. J. Am. Chem. Soc..

[56] Clarke C. J., Gibbard J. A., Hutton L., Verlet J. R. R., Curchod B. F. E. (2022). Photochemistry
of the pyruvate anion produces CO_2_, CO, CH_3_
^–^, CH_3_, and a low energy electron. Nat. Commun..

[57] Cao W., Hu Z., Peng X., Sun H., Sun Z., Wang X.-B. (2022). Annihilating
Actinic Photochemistry of the Pyruvate Anion by One and Two Water
Molecules. J. Am. Chem. Soc..

[58] Miller G. B., Uggerud E. (2017). The Unimolecular Dissociation
of the Glyoxylate, Pyruvate,
Trifluoropyruvate, and α-Oxobutyrate Anions. Decarboxylation
vs. Decarbonylation of Simple α-Ketocarboxylates. Int. J. Mass Spectrom..

[59] Thøgersen J., Madzharova F., Weidner T., Jensen F. (2024). Aqueous pyruvate
partly
dissociates under deep ultraviolet irradiation but is resilient to
near ultraviolet excitation. Nat. Commun..

[60] Weber J. M., Czaplinski E. C., Henderson B. L., Barge L. M., Castillo-Rogez J. C., Hodyss R. (2024). Photochemical Stability and Reactivity of Sodium Pyruvate:
Implications for Organic Analysis on Ceres. ACS Earth Space Chem..

[61] Bersenkowitsch N. K., Madlener S., Heller J., van der Linde C., Ončák M., Beyer M. K. (2023). Spectroscopy of
Cluster Aerosol Models:
IR and UV Spectra of Hydrated Glyoxylate with and without Sea Salt. Environ. Sci.: Atmos..

[62] Herburger A., van der Linde C., Beyer M. K. (2017). Photodissociation spectroscopy of
protonated leucine enkephalin. Phys. Chem. Chem.
Phys..

[63] Dunbar R.
C. (2004). BIRD (Blackbody
Infrared Radiative Dissociation): Evolution, Principles, and Applications. Mass Spectrom. Rev..

[64] Frisch, M. J. ; Trucks, G. W. ; Schlegel, H. B. ; Scuseria, G. E. ; Robb, M. A. ; Cheeseman, J. R. ; Scalmani, G. ; Barone, V. ; Petersson, G. A. ; Nakatsuji, H. ; Li, X. ; Caricato, M. ; Marenich, A. V. ; Bloino, J. ; Janesko, B. G. ; Gomperts, R. ; Mennucci, B. ; Hratchian, H. P. ; Ortiz, J. V. ; Izmaylov, A. F. ; Sonnenberg, J. L. ; Williams-Young, D. ; Ding, F. ; Lipparini, F. ; Egidi, F. ; Goings, J. ; Peng, B. ; Petrone, A. ; Henderson, T. ; Ranasinghe, D. ; Zakrzewski, V. G. ; Gao, J. ; Rega, N. ; Zheng, G. ; Liang, W. ; Hada, M. ; Ehara, M. ; Toyota, K. ; Fukuda, R. ; Hasegawa, J. ; Ishida, M. ; Nakajima, T. ; Honda, Y. ; Kitao, O. ; Nakai, H. ; Vreven, T. ; Throssell, K. ; Montgomery, J. A., Jr ; Peralta, J. E. ; Ogliaro, F. ; Bearpark, M. J. ; Heyd, J. J. ; Brothers, E. N. ; Kudin, K. N. ; Staroverov, V. N. ; Keith, T. A. ; Kobayashi, R. ; Normand, J. ; Raghavachari, K. ; Rendell, A. P. ; Burant, J. C. ; Iyengar, S. S. ; Tomasi, J. ; Cossi, M. ; Millam, J. M. ; Klene, M. ; Adamo, C. ; Cammi, R. ; Ochterski, J. W. ; Martin, R. L. ; Morokuma, K. ; Farkas, O. ; Foresman, J. B. ; Fox, D. J. Gaussian 16, revision A.03; 2016.

[65] Chai J.-D., Head-Gordon M. (2008). Long-range
Corrected Hybrid Density Functionals with
Damped Atom-Atom Dispersion Corrections. Phys.
Chem. Chem. Phys..

[66] Dunning T. H. (1989). Gaussian
basis sets for use in correlated molecular calculations. I. The atoms
boron through neon and hydrogen. J. Chem. Phys..

[67] Schöpfer, G. ; Hütter, M. ; Gatt, M. ; Ončák, M. Genetic Algorithms for Finding Molecules. https://git.uibk.ac.at/c7441332/genetic-algorithms.

[68] Bannwarth C., Ehlert S., Grimme S. (2019). GFN2-xTB-An
Accurate and Broadly
Parametrized Self-Consistent Tight-Binding Quantum Chemical Method
with Multipole Electrostatics and Density-Dependent Dispersion Contributions. J. Chem. Theory Comput..

[69] Neese F. (2025). Software Update:
The ORCA Program System—Version 6.0. Wiley Interdiscip. Rev.:Comput. Mol. Sci..

[70] Weigend F., Ahlrichs R. (2005). Balanced basis sets of split valence, triple zeta valence
and quadruple zeta valence quality for H to Rn: Design and assessment
of accuracy. Phys. Chem. Chem. Phys..

[71] Lehtola S., Steigemann C., Oliveira M. J., Marques M. A. (2018). Recent developments
in libxc—A comprehensive library of functionals for density
functional theory. SoftwareX.

[72] Lu T., Chen F. (2012). Multiwfn: a multifunctional
wavefunction analyzer. J. Comput. Chem..

[73] Lu T. (2024). A comprehensive
electron wavefunction analysis toolbox for chemists, Multiwfn. J. Chem. Phys..

[74] Mekic M., Brigante M., Vione D., Gligorovski S. (2018). Exploring
the ionic strength effects on the photochemical degradation of pyruvic
acid in atmospheric deliquescent aerosol particles. Atmos. Environ..

[75] Samanta B. R., Fernando R., Rösch D., Reisler H., Osborn D. L. (2021). Primary
photodissociation mechanisms of pyruvic acid on S_1_: observation
of methylhydroxycarbene and its chemical reaction in the gas phase. Phys. Chem. Chem. Phys..

[76] Cohen H., Meyerstein D. (1972). Mechanism of reduction of cobalt­(III) and ruthenium­(III)
hexaammine complexes by several aliphatic radicals. J. Am. Chem. Soc..

[77] Rao P. S., Hayon E. (1974). Redox potentials of free radicals. I. Simple organic radicals. J. Am. Chem. Soc..

[78] Russell G. A., Stephens R. D., Talaty E. R. (1965). Application of E.S.R. spectroscopy
to problems of structure and conformation. III. A new method for preparation
of radical-anions of diketones. Tetrahedron
Lett..

[79] Laroff G. P., Fessenden R. W. (1971). ^13^C Hyperfine Interactions in Radicals from
Some Carboxylic Acids. J. Chem. Phys..

[80] Verma N. C., Fessenden R. W. (1976). Time Resolved
ESR Spectroscopy. IV. Detailed Measurement
and Analysis of the ESR Time Profile. J. Chem.
Phys..

[81] Calle P., Sanchez A., Sieiro C. (1991). A study of
the alkaline degradation
of carbohydrates in methyl sulfoxide by e.s.r. spectroscopy: Part
2, monosaccharides. Carbohydr. Res..

[82] Habteyes T., Velarde L., Sanov A. (2006). Solvent-Enabled
Photodissociation
of CO_2_
^–^ in Water Clusters. Chem. Phys. Lett..

