# Supplementary material for: Photochemistry of Sodium Pyruvate Clusters
Source: J Phys Chem A. 2026 Jun 16;130(26):4995–5004. doi: 10.1021/acs.jpca.6c02410 (PMC13339629; doi:10.1021/acs.jpca.6c02410)
Supplement: Supplementary file 1 [file jp6c02410_si_001.pdf]

Supporting Information

Photochemistry of Sodium Pyruvate Clusters

Sarah J. Madlener, Marc Reimann, Jessica C. Hartmann, Christian van der Linde, Milan Ončák and Martin K. Beyer

Universität Innsbruck, Institut für Ionenphysik und Angewandte Physik, Technikerstraße 25, 6020 Innsbruck, Austria

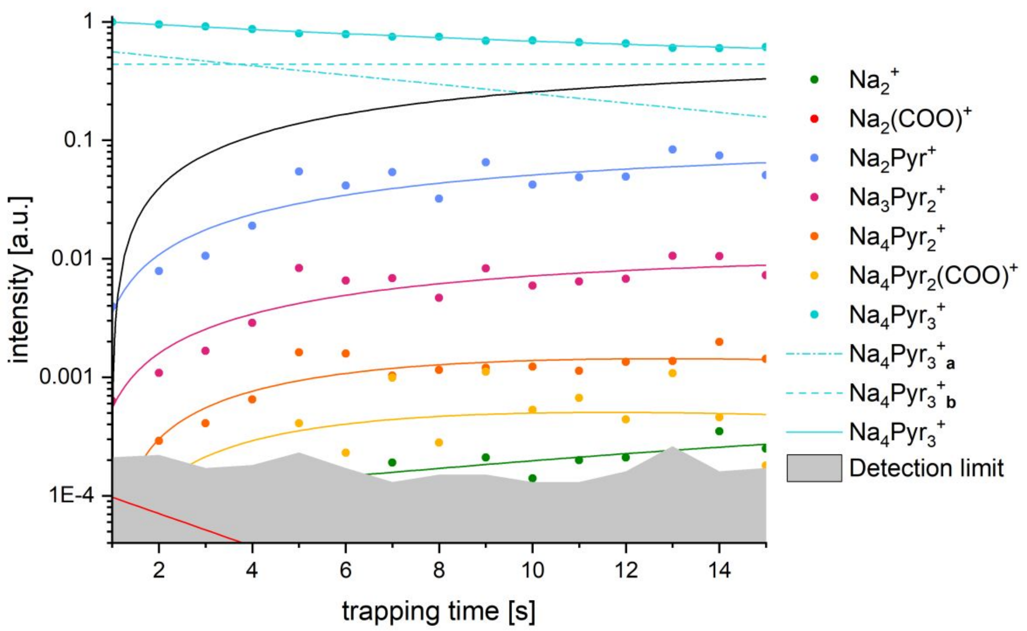

Figure S1: Photodissociation kinetics of $Na_4Pyr_3^+$ at 345 nm with an irradiation time of 1–15 s. The dashed lines represent the reactive and non-reactive fractions of the reactant ion population a and b, respectively. The dark channel accounts for the loss of the stoichiometric Na^+ fragment. Na_2COO^+ has not been observed in the experiment, but it has been added to the kinetics for consistency with the fit in Figure S3 (solid line below the detection limit, no data points observed). The kinetics indicates that $Na_4Pyr_2^+$ is formed as a secondary product.

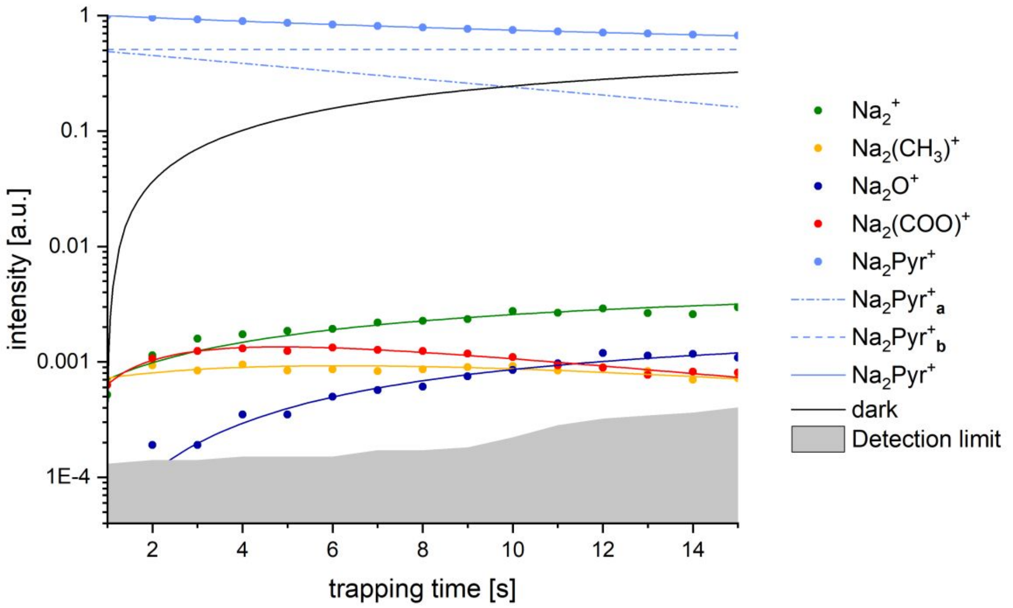

Figure S2: Photodissociation kinetics of Na_2Pyr^+ at 318 nm with an irradiation time of 1–15s. The dashed lines represent the reactive and non-reactive fractions of those reactant ion population a and b, respectively. The dark channel accounts for the loss of the stoichiometric Na^+ fragment.

Table S1: Fit Parameter Matrix for the photodissociation kinetic of $Na_4Pyr_3^+$ shown in Figure S2. Entry "0.00000" indicates that the parameter was active and optimized to zero. Entry "0" denotes an inactive parameter, because the underlying reaction can be ruled out.

Initial Intensities	0.000115	0.000097	0.003476	0.000546	0.000000	0.000000	0.557551	0.438216	0
	Na_2^+	$Na_2CO_2^+$	Na_2Pyr^+	$Na_3Pyr_2^+$	$Na_4Pyr_2^+$	$Na_4Pyr_2CO_2^+$	$Na_4Pyr_3^+{}_a$	$Na_4Pyr_3^+{}_b$	dark
Na_2^+	0	0	0.000001	0.000744	0.000314	0.016947	0.000000	0	0
$Na_2CO_2^+$	0	0	0.000000	0.000000	0	0.000000	0.000000	0	0
Na_2Pyr^+	0	0	0	0.001743	0	0	0.013780	0	0
$Na_3Pyr_2^+$	0	0	0	0	0.073508	0	0.001981	0	0
$Na_4Pyr_2^+$	0	0	0	0	0	0.041828	0.000586	0	0
$Na_4Pyr_2CO_2^+$	0	0	0	0	0	0	0.000231	0	0
$Na_4Pyr_3^+{}_a$	0	0	0	0	0	0	0	0	0
$Na_4Pyr_3^+{}_b$	0	0	0	0	0	0	0	0	0
Na^+ (dark)	0.000023	0.317428	0.000000	0.017874	0.018741	0.038105	0.073902	0	0

Table S2: Fit Parameter Matrix for the photodissociation kinetic of Na_2Pyr^+ shown in Figure S3. Entry "0.00000" indicates that the parameter was active and optimized to zero. Entry "0" denotes an inactive parameter, because the underlying reaction can be ruled out.

Initial Intensities	0.000704	0.000725	0.000046	0.000635	0.489293	0.508596	0
	Na_2^+	$Na_2CH_3^+$	Na_2O^+	$Na_2CO_2^+$	$Na_2Pyr^+{}_a$	$Na_2Pyr^+{}_b$	dark
Na_2^+	0	0.000000	0	0.000000	0.000593	0	0
$Na_2CH_3^+$	0	0	0	0	0.000392	0	0
Na_2O^+	0	0	0	0.075290	0	0	0
$Na_2CO_2^+$	0	0	0	0	0.001629	0	0
$Na_2Pyr^+{}_a$	0	0	0	0	0	0	0
$Na_2Pyr^+{}_b$	0	0	0	0	0	0	0
Na^+ (dark)	0	0.137347	0	0.360908	0.076419	0	0

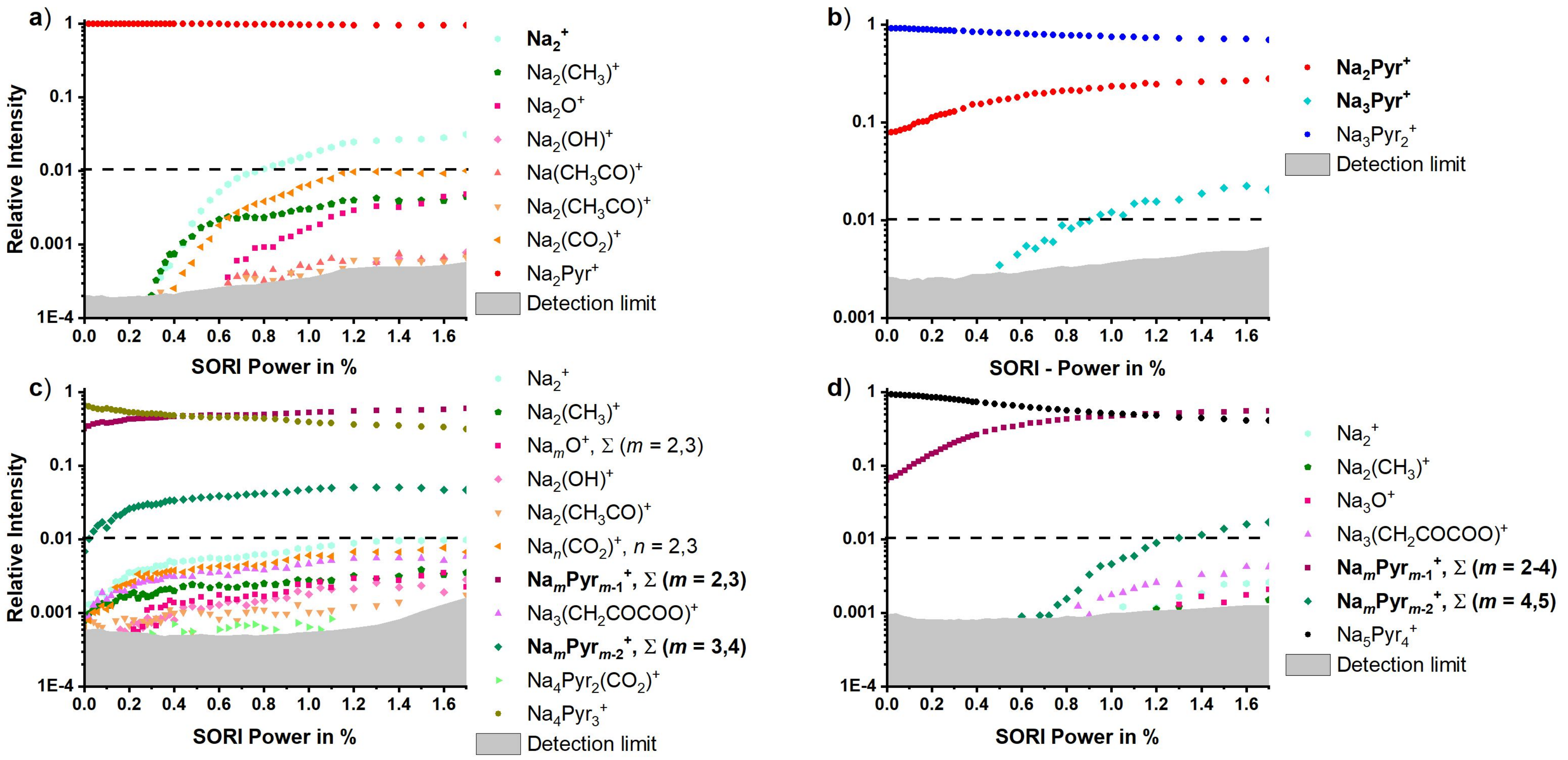

Figure S3: SORI CID spectra of $Na_nPyr_{n-1}^+$, n = 2–5 with increasing n from a) to d). Main fragments are written in a bold font and a limit of 1% fragment intensity has been indicated using a dashed line.

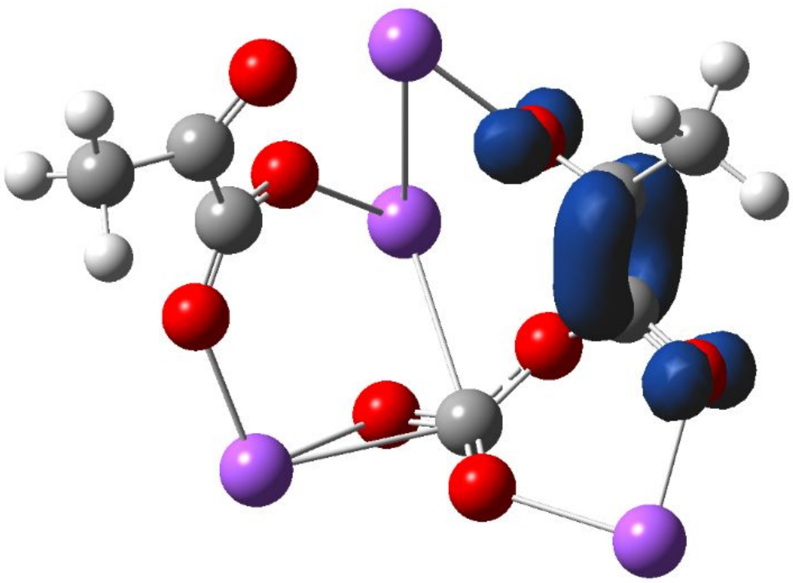

Figure S4: Spin density of $Na_4Pyr_2(CO_2)^+$ iso 1, calculated using ωB97XD/aug-cc-pVDZ level of theory

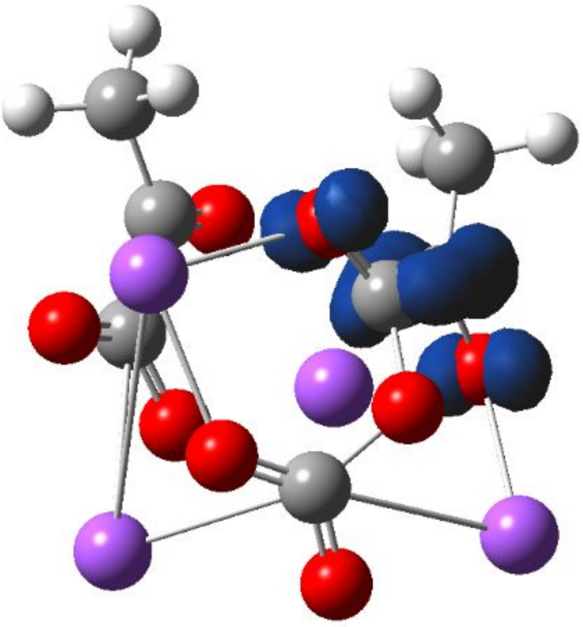

Figure S5: Spin density of $Na_4Pyr_2(CO_2)^+$ iso 2, calculated using ωB97XD/aug-cc-pVDZ level of theory

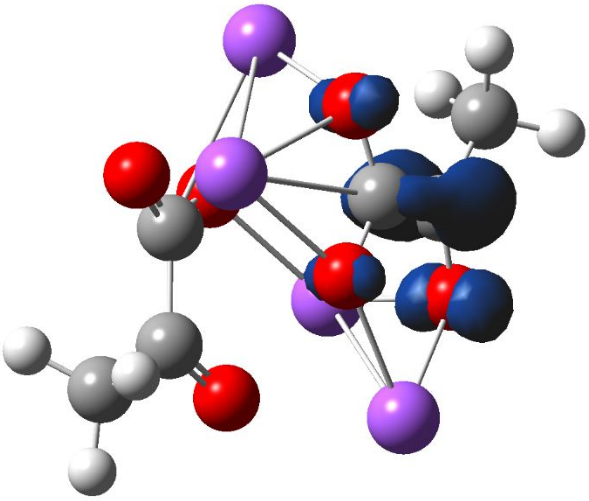

Figure S6: Spin density of $Na_4Pyr_2^+$, calculated using ωB97XD/aug-cc-pVDZ level of theory

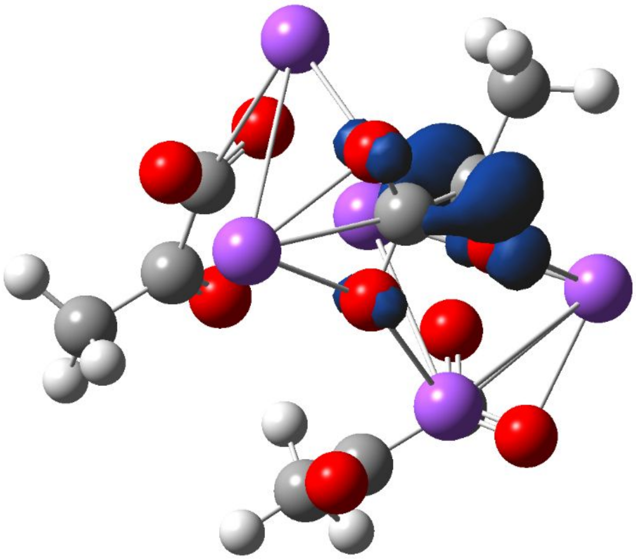

Figure S7: Spin density of $Na_5Pyr_3^+$, calculated using ωB97XD/aug-cc-pVDZ level of theory

Table S3: Natural charges for the structures shown in Figures S4–S7. The units describe different pyruvate molecules, which are separated further into the respective smaller chemical elements. All calculations were conducted using the ωB97XD/aug-cc-pVDZ level of theory.

	CH_3	CO	COO^-	CO_2[a]	Sum
$Na_4Pyr_2^+$ unit 1	-0.023	-0.678	-1.201		-1.902
unit 2	0.040	-0.109	-0.890		-0.959
$Na_5Pyr_3^+$ unit 1	0.050	-0.079	-0.909		-0.938
unit 2	0.042	-0.075	-0.907		-0.940
unit 3	-0.025	-0.716	-1.164		-1.905
$Na_4Pyr_2(CO_2)^+$ iso1 unit 1	0.021	-0.525	-0.771	-0.650	-1.925
unit 2	0.048	-0.101	-0.898		-0.951
$Na_4Pyr_2(CO_2)^+$ iso2 unit 1	0.026	-0.570	-0.762	-0.613	-1.919
unit 2	0.045	-0.085	-0.908		-0.948

[a] *Represents the remaining CO_2 in the cluster after photolysis of another pyruvate molecule*

Computed Structures

Cartesian coordinates of optimized structures (in Ångstrom) along with the respective electronic energy including zero-point energy correction (in Hartree)

Na4Pyr3+ iso I

```
E = -1674.432881

C   1.638111  -4.118286   0.235827
C   1.692962  -2.637036   0.417826
C   0.839578  -1.744045  -0.536711
O   1.059186  -0.507583  -0.473426
O   2.360803  -2.097983   1.281263
O   0.011873  -2.327325  -1.265181
H   0.607363  -4.464602   0.393023
H   2.318552  -4.611190   0.935132
H   1.892186  -4.370277  -0.802720
C  -5.179965  -0.231194   0.614716
C  -4.060092  -0.761442  -0.216881
C  -2.918939   0.207974  -0.629912
O  -2.307320  -0.087648  -1.688282
O  -3.974454  -1.926433  -0.566111
O  -2.672583   1.157611   0.140845
H  -5.625924   0.640491   0.115269
H  -5.930508  -1.007818   0.784004
H  -4.776108   0.132345   1.569354
C   3.211989   3.909793  -1.221329
C   2.826491   2.748866  -0.365264
C   1.345230   2.606952   0.052930
O   0.471297   2.831584  -0.826636
O   3.605089   1.889165   0.005850
O   1.115915   2.224310   1.226496
H   2.956488   4.842842  -0.697717
H   4.281585   3.879971  -1.445664
H   2.614498   3.898061  -2.142411
Na -2.103588  -2.380111  -1.918266
Na -0.247256   0.845233  -1.787930
Na -1.202669   2.587606   0.928621
Na  2.491237   0.227907   1.275710
```

Na4Pyr3+ iso II

```
E = -1674.428345

C   1.970467   2.201239   2.158049
C   1.969058   1.866260   0.704150
C   0.605839   1.586450   0.012361
O   0.542046   1.844139  -1.220199
O   2.984769   1.776039   0.036041
O  -0.317261   1.121603   0.716373
H   1.187846   2.936788   2.382753
H   2.956401   2.565074   2.459609
H   1.726682   1.289095   2.720659
Na -1.212001  -2.374835  -1.448352
Na  2.556945   1.072032  -2.164275
Na -0.372930  -1.076197   1.529663
Na -1.813565   1.747575  -1.333877
C  -4.824536  -0.063377   1.444189
C  -3.813839   0.399428   0.448184
C  -2.862206  -0.651973  -0.173890
O  -2.443071  -1.574538   0.567461
O  -3.676732   1.561079   0.109163
O  -2.534891  -0.471889  -1.376245
H  -5.467963  -0.823701   0.977716
H  -5.423858   0.778869   1.799910
H  -4.313002  -0.563135   2.277359
C   4.108937  -1.459089   0.752620
C   2.619886  -1.377028   0.804857
C   1.817834  -1.509563  -0.518468
O   2.348113  -1.058317  -1.549754
O   1.997776  -1.155993   1.831961
O   0.672292  -2.013583  -0.396545
H   4.423508  -2.303644   0.126469
H   4.520328  -1.537820   1.762711
H   4.485064  -0.546054   0.269717
```

Na4Pyr3+ iso III

```
E = -1674.427377

C   4.540491  -1.693939   0.243860
C   3.559913  -0.566923   0.252263
C   2.311748  -0.661328  -0.665060
O   1.820974   0.422715  -1.050805
O   3.679351   0.418505   0.958327
O   1.867454  -1.808249  -0.920810
H   4.046398  -2.609618   0.595805
H   5.398579  -1.456060   0.878089
H   4.861167  -1.892868  -0.788323
C  -1.668517   3.990423  -0.958559
C  -0.503852   3.246218  -0.392697
C  -0.729487   1.800520   0.104159
O  -0.183830   1.502196   1.201765
O   0.625340   3.704482  -0.332244
O  -1.376815   1.047794  -0.653061
H  -2.070976   3.431940  -1.814021
H  -1.370297   4.999272  -1.256218
H  -2.471382   4.031272  -0.208066
C  -4.165178  -1.105479  -0.734777
C  -3.138454  -1.096441   0.346756
C  -1.801369  -1.851000   0.088661
O  -1.535992  -2.197644  -1.091521
O  -3.282080  -0.521452   1.408425
O  -1.054388  -2.051239   1.081732
H  -3.768238  -0.538371  -1.588215
H  -5.091792  -0.648189  -0.378130
H  -4.337304  -2.129815  -1.089233
Na -1.206467  -0.150030   2.390365
Na  2.122998   2.152562   0.561980
Na -0.199568  -0.444530  -1.917922
Na  0.398553  -3.322890  -0.282453
```

Na5Pyr4+ iso I
E =-2178.549216

C	0.659702	0.812581	-3.865832
C	0.310749	1.487877	-2.581831
C	-0.963107	1.034196	-1.816908
O	-1.300091	-0.152709	-1.970617
O	1.025166	2.345855	-2.075892
O	-1.496777	1.904963	-1.083446
H	1.184129	-0.119226	-3.606385
H	1.333714	1.443683	-4.453027
H	-0.240034	0.548934	-4.432112
C	4.302479	-2.434940	1.084772
C	3.734583	-1.329498	0.256723
C	2.339599	-1.552140	-0.396905
O	1.663420	-2.529411	0.007116
O	4.299708	-0.266540	0.085482
O	1.977942	-0.731781	-1.277459
H	3.675210	-2.559637	1.978607
H	5.328269	-2.196944	1.378138
H	4.254417	-3.383796	0.535480
C	-0.674284	1.023022	3.821039
C	-0.340375	1.630073	2.499482
C	0.940373	1.157565	1.758575
O	1.461388	1.998338	0.982056
O	-1.071167	2.444811	1.947468
O	1.295422	-0.014743	1.971436
H	0.231319	0.809997	4.399203
H	-1.359553	1.673577	4.372910
H	-1.180558	0.067735	3.616975
C	-4.235925	-2.533602	-0.989756
C	-3.701733	-1.386686	-0.196389
C	-2.311041	-1.555648	0.482428
O	-1.619292	-2.545593	0.139664
O	-4.290843	-0.330283	-0.072298
O	-1.967820	-0.683727	1.319451
H	-5.265070	-2.331187	-1.297804
H	-4.167493	-3.462473	-0.409672
H	-3.599783	-2.669811	-1.875733
Na	-0.114273	-2.050763	-1.545732
Na	-0.030077	3.339490	-0.095070
Na	0.146298	-1.957575	1.662251
Na	-2.886110	1.359127	0.659300
Na	2.858020	1.358387	-0.720839

Na5Pyr4+ iso II
E =-2178.546257

C	-3.835176	0.860407	-1.106306
C	-2.905270	-0.301764	-1.024697
C	-1.614822	-0.283163	-1.886539
O	-1.100662	0.826683	-2.111969
O	-3.088647	-1.264657	-0.293841
O	-1.150674	-1.404805	-2.218166
H	-4.346941	0.812822	-2.080141
H	-4.573981	0.823318	-0.301429
H	-3.269801	1.798750	-1.106153
C	-2.824124	3.700550	1.648486
C	-1.730523	3.145735	0.794671
C	-1.126851	1.767995	1.187781
O	0.055421	1.556787	0.820785
O	-1.314627	3.706144	-0.203832
O	-1.884314	0.989714	1.804241
H	-3.670753	3.001365	1.647047
H	-3.129939	4.684796	1.283945
H	-2.480216	3.761199	2.690480
Na	-2.048100	-1.213841	2.067941
Na	0.278786	2.463430	-1.411761
Na	-1.595697	-3.247874	-1.015246
Na	1.103910	-0.884340	-2.212201
Na	1.553378	0.162817	1.887970
C	4.649556	2.483268	-0.434335
C	3.612948	1.616211	0.201562
C	2.700378	0.771481	-0.718477
O	2.408056	-0.381670	-0.314791
O	3.426828	1.552674	1.403647
O	2.272567	1.312360	-1.768188
H	5.321003	1.856493	-1.039360
H	5.215864	3.024355	0.328339
H	4.165509	3.178192	-1.133025
C	2.619863	-3.379365	0.858950
C	1.234435	-3.155986	0.355509
C	0.149456	-2.660697	1.339631
O	-1.012762	-3.105261	1.165186
O	0.909925	-3.292967	-0.815619
O	0.483218	-1.795171	2.178748
H	3.093568	-2.392989	0.966020
H	3.191161	-3.974759	0.140849
H	2.603035	-3.853216	1.847828

Na5Pyr4+ iso III
E = -2178.546257

C	4.757294	2.220922	0.364483
C	3.856979	1.086475	0.000908
C	2.422838	1.420271	-0.488898
O	1.874647	0.591017	-1.256261
O	4.179479	-0.083723	0.086496
O	1.896885	2.477597	-0.061126
H	4.834362	2.914435	-0.484803
H	5.743876	1.846605	0.649827
H	4.304919	2.792821	1.185558
C	0.432863	-0.771185	-3.879365
C	0.046178	-1.445601	-2.605065
C	-1.157244	-0.889398	-1.791505
O	-1.741659	-1.718644	-1.051868
O	0.674482	-2.391621	-2.146007
O	-1.390778	0.326492	-1.919622
H	1.049651	0.098765	-3.610238
H	-0.449532	-0.409161	-4.418269
H	1.032656	-1.447061	-4.496172
C	-4.055148	2.635596	-0.910417
C	-3.530955	1.567426	-0.008035
C	-2.109786	1.758877	0.598024
O	-1.427660	2.731324	0.184587
O	-4.150000	0.557251	0.261338
O	-1.728701	0.923973	1.455412
H	-3.458539	2.625469	-1.833661
H	-5.105338	2.445031	-1.146781
H	-3.919949	3.623878	-0.454204
C	-0.064915	-0.673943	3.875202
C	0.541613	-0.870482	2.524091
C	0.149291	-2.131461	1.727151
O	1.025945	-2.651680	0.996228
O	1.271072	-0.048952	1.990700

O	-1.045530	-2.496295	1.819584
H	0.487803	0.087953	4.434047
H	-0.100354	-1.617784	4.431652
H	-1.103085	-0.342726	3.727542
Na	-0.053055	2.111112	-1.552333
Na	0.375469	2.167135	1.636934
Na	-0.589026	-3.582674	-0.407633
Na	2.408277	-1.547839	-0.397246
Na	-2.737772	-1.146356	0.983911

Na3Pyr2+

E = -1170.317461

C	3.181128	1.670229	-0.763655
C	2.246160	0.975732	0.167808
C	1.705095	-0.421283	-0.215501
O	1.326651	-0.587718	-1.396414
O	1.847890	1.459756	1.215557
O	1.608871	-1.249838	0.727498
H	2.708102	1.772009	-1.749192
H	3.465930	2.647391	-0.364623
H	4.070864	1.040413	-0.911473
C	-3.183836	1.670040	0.757298
C	-2.247183	0.973742	-0.171107
C	-1.704381	-0.421176	0.217339
O	-1.325767	-0.582788	1.398840
O	-1.848926	1.454749	-1.220264
O	-1.607135	-1.253039	-0.722666
H	-4.072706	1.039364	0.906660
H	-3.469873	2.645382	0.354714
H	-2.711637	1.775922	1.742794
Na	0.001410	-2.679643	0.004068
Na	-0.580373	-0.068572	-2.488014
Na	0.581120	-0.060076	2.488769

Na2Pyr+

E = -666.203474

Na	1.562443	-2.004232	0.000000
Na	-2.839379	-0.576451	0.000000
C	1.153098	2.447308	0.000000
C	1.053339	0.962523	0.000000
C	-0.369122	0.326829	0.000000
O	-0.437852	-0.944046	0.000000
O	2.021153	0.219405	0.000000
O	-1.343457	1.097986	0.000000
H	0.622566	2.845902	0.876073
H	2.200365	2.759924	0.000000
H	0.622566	2.845902	-0.876073

Na4Pyr2CO2+ iso I

E = -1521.204683

C	-1.798345	-3.222257	0.857683
C	-1.517061	-2.031944	-0.014498
C	-2.384333	-0.899401	0.070699
O	-3.348681	-0.747387	0.842065
O	-0.498350	-2.038301	-0.795578
O	-2.078392	0.089537	-0.885288
H	-1.788775	-4.132750	0.243552
H	-2.763431	-3.135644	1.365118
H	-1.013466	-3.327533	1.623498
C	4.555156	0.669512	1.415981
C	3.475594	-0.140729	0.780001
C	2.433482	0.588602	-0.115659
O	1.879366	-0.123883	-0.991361
O	3.350723	-1.342538	0.952800
O	2.218108	1.786441	0.151387
H	5.097545	1.227259	0.639456
H	5.235590	0.025571	1.979556
H	4.097886	1.423516	2.070644
O	-0.940116	1.882775	-1.416811
C	-1.625698	1.345499	-0.517341
O	-1.880131	1.809076	0.615475
Na	0.459349	3.099541	-0.140656
Na	1.649483	-2.340262	-0.283275
Na	-0.058193	-0.189025	-2.172861
Na	-3.723959	1.211085	1.738856

Na4Pyr2CO2+ iso II

E = -1521.200467

C	0.760702	-2.696431	1.325769
C	-0.325043	-1.831502	0.753179
C	-0.603796	-0.566454	1.368429
O	0.125385	0.046001	2.163821
O	-0.984275	-2.231070	-0.284861
O	-1.847520	-0.035336	0.975104
H	1.483301	-2.991563	0.552695
H	0.309259	-3.618912	1.717366
H	1.291673	-2.192152	2.138572
C	3.467288	0.063531	0.740320
C	2.573675	-0.243847	-0.414439
C	1.557904	0.846353	-0.873966
O	0.731557	0.504646	-1.762371
O	2.575801	-1.316322	-0.989863
O	1.623232	1.968905	-0.326872
H	4.229204	-0.713305	0.846197
H	2.836894	0.079132	1.643975
H	3.918066	1.056477	0.630530
O	-2.551591	0.946964	-0.886686
C	-1.871587	1.099726	0.148875
O	-1.225097	2.105990	0.492926
Na	0.529349	-1.790724	-1.977255
Na	-3.039947	-1.312417	-0.583272
Na	-0.553645	2.468541	-1.689268
Na	0.613033	2.210816	1.774607

Na2CO2+ iso I

E = -512.958513

C	-0.003656	-0.393704	0.000000
O	1.131352	0.096117	0.000000
Na	3.235078	0.033659	0.000000
O	-1.129238	0.117576	0.000000
Na	-3.234550	0.028531	0.000000

Na2CO2+ iso II

E = -512.955092

C	1.250619	0.709767	1.424044
O	1.778398	1.842673	1.608393
O	0.721735	0.210117	0.444667
Na	2.385252	1.611601	3.736168
Na	1.029433	2.014578	-0.877321

Na5Pyr3+ iso I

E = -1836.763282

C	-3.820750	2.253112	-0.264209
C	-3.046817	1.201647	0.455262
C	-3.128593	-0.258312	-0.035182
O	-2.891612	-0.470393	-1.254439
O	-2.296167	1.421174	1.391932
O	-3.290221	-1.135766	0.847881
H	-4.885407	1.980241	-0.279051
H	-3.677165	3.226849	0.211775
H	-3.483233	2.280183	-1.308872
C	0.944256	3.731773	0.027472
C	0.663370	2.431614	-0.653295
C	1.845061	1.447452	-0.886712
O	1.642273	0.515699	-1.705933
O	-0.448399	2.097099	-1.014262
O	2.898497	1.633098	-0.230450
H	1.192998	3.531965	1.079758
H	0.064657	4.378932	-0.021684
H	1.822764	4.215072	-0.417648
Na	3.013090	0.749378	1.909613
Na	-0.598981	-0.169290	-1.633847
Na	-1.160188	-0.639442	1.938662
Na	-2.440340	-2.766338	-0.522941
Na	3.564505	-0.650852	-1.143273
C	1.503528	-3.167764	-0.779453
C	0.847831	-2.122482	0.075833
C	1.639771	-1.253424	0.931644
O	1.047400	-0.388071	1.679939
O	-0.446713	-1.939466	-0.030483
O	2.926420	-1.309138	0.879608
H	1.538247	-2.858884	-1.842220
H	0.927566	-4.104049	-0.749907
H	2.522865	-3.383912	-0.441011

Na5Pyr3+ iso II

E = -1836.761478

C	0.491828	5.246424	-0.498857
C	-0.217156	3.958784	-0.232703
C	0.638163	2.703979	0.117246
O	0.024506	1.770275	0.687846
O	-1.427328	3.839040	-0.296692
O	1.837231	2.736062	-0.227043
H	1.142378	5.490301	0.352485
H	-0.230399	6.047119	-0.678903
H	1.154752	5.119735	-1.365330
C	3.096851	-0.355344	1.649168
C	2.818347	-1.133786	0.386387
C	1.761221	-2.136260	0.373987
O	0.973047	-2.214286	1.386309
O	3.520238	-0.858920	-0.691312
O	1.593389	-2.896100	-0.654798
H	4.178078	-0.176649	1.743825
H	2.765151	-0.903845	2.537682
H	2.596512	0.634287	1.665646
Na	3.490798	1.372383	-0.679116
Na	-0.526427	-3.494267	0.237144
Na	-0.035480	-0.254847	1.680133
Na	3.296867	-2.639467	-2.012996
C	-5.127804	-1.697708	-0.934921
C	-4.066900	-0.740377	-0.502204
C	-2.773442	-1.319413	0.144304
O	-2.535820	-2.524612	-0.053577
O	-4.152690	0.466298	-0.650308
O	-2.072577	-0.489376	0.781713
H	-4.707656	-2.387749	-1.678933
H	-5.985942	-1.157404	-1.343481
H	-5.427576	-2.319643	-0.079822
Na	-2.350800	1.740598	0.216971

Na4Pyr2+

E = -1332.651573

C	3.148011	0.191505	-0.349076
C	1.955736	-0.375330	0.373511
C	1.015844	0.508125	1.040231
O	0.010308	0.011044	1.691356
O	1.733010	-1.660428	0.293583
O	1.130217	1.786894	0.898116
H	2.983670	0.232636	-1.446030
H	4.017073	-0.461881	-0.199907
H	3.396696	1.199343	0.001902
C	-3.249808	-0.574853	0.261025
C	-1.947806	-0.793828	-0.440297
C	-1.221658	0.439584	-1.046777
O	-1.556582	1.563415	-0.610750
O	-1.418060	-1.892560	-0.512760
O	-0.304655	0.206832	-1.874499
H	-3.038067	-0.138038	1.248202
H	-3.771202	-1.526989	0.396648
H	-3.869686	0.145902	-0.284374
Na	0.692461	-1.845560	-1.675726
Na	0.111439	-2.209145	1.707583
Na	-1.007979	2.068090	1.614747
Na	0.844953	2.190978	-1.280316

Na3(CH2COCOO)+

E = -827.940143

C	1.241140	-1.648116	-0.921216
C	0.150927	-1.186850	-0.216465
C	-0.018996	0.341392	-0.108874
O	1.005567	1.085395	0.050669
O	-0.752830	-1.871796	0.368513
O	-1.193135	0.822646	-0.147481
H	1.348530	-2.716073	-1.102544
H	1.804985	-0.959781	-1.555660
Na	-0.328486	2.928716	-0.193979

Na	2.688195	-0.351490	0.695662
Na	-2.686210	-0.947299	0.247876

Na2(CH3CO)+ iso I

E = -477.611270

C	-2.467788	0.707837	0.125449
C	-1.269057	0.271706	0.581480
O	-0.329349	-0.317529	-0.148688
Na	0.206084	-2.346525	0.239831
Na	-0.437240	1.497014	-1.470204
H	-2.820181	0.461651	-0.882129
H	-3.160692	1.212620	0.794484
H	-1.019046	0.462777	1.639271

Na2(CH3CO)+ iso II

E = -477.572137

Na	-2.626685	0.384798	0.000168
Na	2.634709	0.901077	-0.000512
C	0.250534	-1.774172	0.000150
C	0.528360	-0.284667	-0.000025
O	-0.489968	0.451447	0.000005
H	0.732812	-2.232127	0.878157
H	-0.828306	-2.024414	0.002232
H	0.728943	-2.230857	-0.880690

Na(CH3CO)+ iso I

E = -315.223826

C	-2.553876	0.091074	0.264629
C	-1.520744	1.058353	0.400586
O	-0.436181	0.970742	-0.203372
Na	1.538333	1.146489	-1.075723
H	-2.412149	-0.769569	-0.389067
H	-3.489129	0.204190	0.811356
H	-1.698891	1.916626	1.070635

Na(CH3CO)+ iso II

E = -315.223281

C	-1.750499	0.711018	0.001109
C	-0.884940	-0.487968	-0.146506
Na	-0.765998	-3.816840	-0.526872
O	-1.171767	-1.642947	-0.274605
H	-1.474642	1.216358	0.936705
H	-2.815688	0.442646	-0.007895
H	-1.508422	1.405098	-0.815520

Na2CH3+ iso I

E = -364.260323

C	0.000000	0.000000	0.074146
Na	0.000000	0.000000	2.463354
Na	0.000000	0.000000	-2.435296
H	0.000000	1.069480	-0.192746
H	0.926197	-0.534740	-0.192746
H	-0.926197	-0.534740	-0.192746
XX	1.000000	0.000000	0.074146

Na2CH3+ iso II

E = -364.252393

C	0.075849	-0.051291	0.110269
Na	-0.803970	0.043278	2.344252
Na	2.417549	0.019779	0.449669
H	-1.025765	-0.082506	-0.008137
H	0.331596	0.807112	-0.545280
H	0.371894	-0.959643	-0.454772

Na4Pyr4

E = -2016.374738

C	5.350174	-1.577861	-0.225640
C	3.868616	-1.557951	-0.437102
C	2.984005	-0.995538	0.706336
O	3.501487	-0.133093	1.438640
O	3.339513	-1.918734	-1.474030
O	1.797874	-1.419933	0.738623
H	5.695665	-0.547895	-0.066270
H	5.852073	-2.031792	-1.085008
H	5.581105	-2.125226	0.698835
C	-2.315135	3.568636	1.365933
C	-1.094109	2.720503	1.539652
C	-1.252057	1.180289	1.436027
O	-2.142231	0.757148	0.673843
O	0.020837	3.186738	1.701973
O	-0.421915	0.509841	2.099779
H	-3.133820	3.184075	1.987803
H	-2.089439	4.611247	1.608733
H	-2.644501	3.483085	0.321025
C	-4.156668	-3.834745	0.369608
C	-2.845892	-3.137558	0.559246
C	-2.292130	-2.306296	-0.627743
O	-3.131424	-1.815659	-1.404308
O	-2.226397	-3.159269	1.608554
O	-1.041860	-2.151431	-0.649432
H	-4.926629	-3.081903	0.155131
H	-4.412539	-4.414899	1.260917
H	-4.103468	-4.480375	-0.518025
C	0.359346	4.121761	-1.618140
C	-0.341408	2.803707	-1.727616
C	0.496668	1.511366	-1.538281
O	1.472066	1.582810	-0.766364
O	-1.544028	2.703419	-1.902161
O	0.069621	0.500213	-2.150207
H	0.678594	4.253340	-0.574890
H	-0.311792	4.933081	-1.915226
H	1.271144	4.116565	-2.229293
Na	-2.199465	0.311396	-1.584637
Na	1.693513	1.339296	1.513257
Na	-0.301598	-1.689971	1.528724
Na	0.958852	-1.475361	-1.452400

Na3Pyr3
E = -1512.257286

C	3.456244	-4.165007	-0.675716
C	2.922498	-2.786288	-0.447870
C	1.556501	-2.651173	0.282978
O	0.848012	-3.672484	0.370686
O	3.495058	-1.779692	-0.827996
O	1.260346	-1.496676	0.694302
H	3.543498	-4.683321	0.289348
H	4.422811	-4.121480	-1.185829
H	2.724463	-4.738957	-1.259654
C	-5.546500	-0.567357	-0.608319
C	-4.206092	0.062413	-0.398461
C	-3.110777	-0.785650	0.308868
O	-3.307272	-2.011981	0.408261
O	-3.934055	1.189347	-0.774976
O	-2.086727	-0.157712	0.691942
H	-5.949626	-0.892406	0.360841
H	-5.421608	-1.482005	-1.203183
H	-6.225988	0.134387	-1.100430
C	2.466841	4.808240	0.448630
C	2.234564	3.352498	0.193987
C	0.789697	2.901942	-0.158844
O	-0.138845	3.676789	0.144015
O	3.114876	2.513579	0.278747
O	0.675699	1.761992	-0.685266
H	2.139371	5.386160	-0.426862
H	3.522075	4.994389	0.668184
H	1.825522	5.130611	1.279609
Na	-1.673505	1.880271	-0.218127
Na	-0.981962	-2.243706	1.018694
Na	2.345948	0.275204	-0.222996

Na2Pyr2 iso I
E = -1008.153578

C	4.402010	-0.415247	0.162562
C	2.929514	-0.648498	0.043538
C	2.034716	0.532188	-0.397531
O	2.325470	1.656422	0.063054
O	2.395645	-1.710327	0.326702
O	1.032075	0.236167	-1.098923
H	4.791659	-0.068076	-0.805174
H	4.912909	-1.328720	0.480800
H	4.570213	0.403045	0.875107
C	-4.402025	-0.415261	-0.162638
C	-2.929517	-0.648569	-0.043858
C	-2.034704	0.531910	0.397735
O	-1.031974	0.235544	1.098859
O	-2.395663	-1.710245	-0.327611
O	-2.325532	1.656373	-0.062245
H	-4.570288	0.403373	-0.874776
H	-4.912957	-1.328578	-0.481271
H	-4.791586	-0.068552	0.805299
Na	0.000015	-1.531771	-0.000554
Na	-0.000045	2.144794	0.000394

Na2Pyr2 iso II
E = -1008.149612

C	-3.708701	0.249065	1.538229
C	-2.668821	-0.281897	0.603162
C	-1.828340	0.732540	-0.207638
O	-1.513899	1.796699	0.362506
O	-2.417722	-1.470469	0.484654
O	-1.447159	0.344625	-1.345054
H	-3.220760	0.913656	2.263463
H	-4.229276	-0.571171	2.041206
H	-4.416867	0.873500	0.974894
Na	0.484221	1.740840	-1.011746
Na	-0.484460	-1.740157	-1.011426
C	3.708216	-0.249382	1.538823
C	2.669407	0.281925	0.602774
C	1.828510	-0.732325	-0.207820
O	1.447038	-0.344176	-1.345059
O	2.418629	1.470564	0.484263
O	1.513496	-1.796119	0.362697
H	3.219369	-0.913689	2.263709
H	4.228729	0.570679	2.042149
H	4.416584	-0.874179	0.976143

NaPyr
E = -504.035131

C	-2.039733	-1.033444	-0.000097
C	-0.605562	-0.610177	0.000082
C	-0.303468	0.934099	-0.000058
O	-1.296457	1.659928	0.000068
O	0.308468	-1.430524	0.000079
O	0.926607	1.235220	-0.000141
H	-2.539575	-0.593749	-0.873538
H	-2.122933	-2.124750	-0.000114
H	-2.539761	-0.593757	0.873238
Na	2.327662	-0.371756	0.000030

Pyr
E = -341.646491

C	-1.755910	-1.148009	-0.008177
C	-1.335909	0.300162	-0.000734
C	1.862386	-0.017902	0.000638
O	2.143103	1.109289	-0.008018
O	-2.011348	1.270169	0.008424
O	1.604440	-1.154068	0.009378
H	-1.313189	-1.639307	0.867148
H	-2.850767	-1.241691	-0.001112
H	-1.326225	-1.625875	-0.897399

CH3CHO

E = -153.744006

C	2.135724	-1.101941	0.000064
C	1.371507	0.189967	0.000375
O	1.878448	1.286079	-0.000160
H	1.849001	-1.692157	-0.883216
H	3.215032	-0.916097	0.000036
H	1.849098	-1.692490	0.883151
H	0.259482	0.090214	-0.000268

CH3CO iso I

E = -153.105309

C	-2.292546	0.161212	0.000267
C	-0.923692	-0.474266	0.000669
O	0.134849	0.052742	-0.001146
H	-2.840068	-0.199397	-0.879711
H	-2.220353	1.258269	-0.007419
H	-2.833576	-0.186944	0.889326

CH3CO iso II

E = -153.096259

C	-2.154084	0.234149	-0.264274
C	-0.941307	-0.484288	-0.039622
O	0.109256	0.066688	0.296449
H	-3.065624	-0.284410	-0.561917
H	-2.165288	1.316611	-0.135648
H	-0.972306	-1.585284	-0.180352
